# Engineering yeast for tailored fatty acid profiles

**DOI:** 10.1007/s00253-025-13487-1

**Published:** 2025-04-22

**Authors:** Simon Kobalter, Tamara Wriessnegger, Harald Pichler

**Affiliations:** 1 Austrian Centre of Industrial Biotechnology (acib) GmbH, Petersgasse 14, 8010 Graz, Austria; 2 Institute of Molecular Biotechnology, Graz University of Technology, NAWI Graz, BioTechMed Graz, Petersgasse 14, 8010 Graz, Austria

**Keywords:** Yeast, Lipid, Fatty acid, Metabolic engineering, Cell factory, Renewable resources

## Abstract

**Abstract:**

The demand for sustainable and eco-friendly alternatives to fossil and plant oil-derived chemicals has spurred interest in microbial production of lipids, particularly triacylglycerols, fatty acids, and their derivatives. Yeasts are promising platforms for synthesizing these compounds due to their high lipid accumulation capabilities, robust growth, and *g*enerally *r*ecognized *a*s *s*afe (GRAS) status. There is vast interest in fatty acid and triacylglycerol products with tailored fatty acid chain lengths and compositions, such as polyunsaturated fatty acids and substitutes for cocoa butter and palm oil. However, microbes naturally produce a limited set of mostly long-chain fatty acids, necessitating the development of microbial cell factories with customized fatty acid profiles. This review explores the capabilities of key enzymes involved in fatty acid and triacylglycerol synthesis, including fatty acid synthases, desaturases, elongases, and acyltransferases. It discusses factors influencing fatty acid composition and presents engineering strategies to enhance fatty acid synthesis. Specifically, we highlight successful engineering approaches to modify fatty acid profiles in triacylglycerols and produce tailored fatty acids, and we offer recommendations for host selection to streamline engineering efforts.

**Key points:**

• *Detailed overview on all basic aspects of fatty acid metabolism in yeast*

• *Comprehensive description of fatty acid profile tailoring in yeast*

• *Extensive summary of applying tailored fatty acid profiles in production processes*

**Graphical abstract:**

**Figure:**
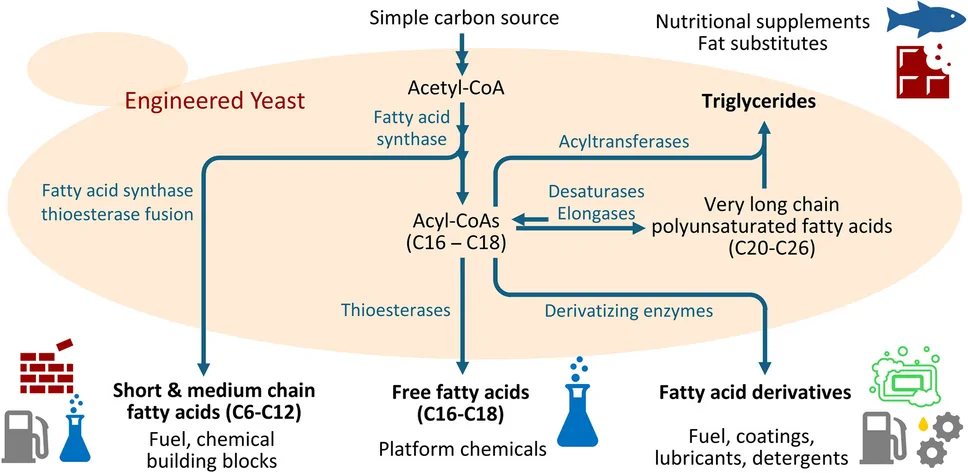

## Introduction

The continued reliance on fossil oils has raised significant environmental and sustainability concerns, yet the demand for oleochemicals continues to grow. Triacylglycerols (TAGs), fatty acids (FAs), and their derivatives can serve as sustainable substitutes for fossil oils, enabling the production of oleochemical-derived products such as fuels, surfactants, lubricants, cosmetics, and pharmaceuticals. Moreover, TAGs are extensively used in the food industry, and high-value FAs find applications as nutritional supplements. Currently, the production of TAGs and FAs primarily relies on extraction from oil-rich plants, with a smaller contribution from animal fat and fish oil. However, the use of plants for oleochemical production processes presents various drawbacks, e.g., competing for farmland with food crops, fluctuating yields due to environmental variations, limiting productivity due to extended lifecycles, and deforestation for new farmland (Meijaard et al. 2020). Notably, extracting oils from fish and animal fats raises sustainability concerns, as extensive animal and fish farming contributes to pollution and increased greenhouse gas emissions.

Microbial production processes, on the other hand, offer a greener, more sustainable, and efficient alternative for synthesizing oleochemical products as reviewed (Liu et al. 2021). Conventional and oleaginous yeast species represent versatile platforms for producing a wide range of bio-based products, including recombinant proteins, peptides, terpenoids, TAGs, FAs, and FA derivatives (Arhar et al. 2021; Cao et al. 2018; Wriessnegger et al. 2014; Xue et al. 2013; Zhou et al. 2016; Zhu et al. 2018). These microorganisms naturally exhibit high lipid accumulation capabilities and robust growth to high cell densities and, in most cases, are classified as *g*enerally *r*ecognized *a*s *s*afe (GRAS) and *q*ualified *p*resumption of *s*afety (QPS) by regulatory agencies, enhancing their industrial appeal. Furthermore, the genomes of many yeast species have been sequenced, lipid synthesis metabolic pathways are well understood, and efficient genome editing tools, including CRISPR/Cas9, along with efficient transformation protocols and strategies for fine-tuned expression of complex metabolic pathways, have been established (Abeln and Chuck 2021; Schindler 2020). Particularly, *Saccharomyces cerevisiae* and *Yarrowia lipolytica* are well-studied regarding lipid storage and have been extensively engineered to increase TAG and FA titers, yields, and productivity (Arhar et al. 2021; Qiao et al. 2017). Consequently, these yeasts serve as hosts and model organisms for studying lipid metabolism.

There is a growing interest in FAs with specific chain lengths and in TAGs with customized FA compositions. FAs with industrial importance include short-chain fatty acids (SCFAs), medium-chain fatty acids (MCFAs), long-chain fatty acids (LCFAs), and very long-chain fatty acids (VLCFAs) with varying degrees of saturation. S/MCFAs (C6–C12) and their derivatives are used as herbicides, antimicrobials, lubricants, plastic monomers, and jet fuel replacements (Sarria et al. 2017). LCFAs (C14–C20) and derivatives find applications in biodiesel, cosmetics, nutritional supplements, and coating materials (Runguphan and Keasling 2014). VLCFAs (C20–C26), especially polyunsaturated types like docosahexaenoic acid (DHA) and eicosapentaenoic acid (EPA), offer various health benefits and have been shown to prevent cancer, diabetes, and cardiovascular diseases (Deckelbaum and Torrejon 2012). Additionally, these FAs function as crucial precursors to signaling compounds in the human body (Funk 2001). The need for customized fats stems from the increasing interest in developing substitutes for cocoa butter and palm oil (Bergenholm et al. 2018; Karamerou et al. 2021). The major fatty acid species found in yeasts typically have lengths of 16 or 18 carbon atoms with up to three double bonds and smaller fractions of C12, C14, and C20–C26 species (Kaneko et al. 1976).

Given the restricted set of FA species produced by wild-type yeasts and the ongoing demand in FAs and TAGs with tailored chain lengths and compositions, there is a significant interest in engineering yeasts for optimized fatty acid profiles (Bergenholm et al. 2018; Xue et al. 2013; Zhu et al. 2020). Moreover, commercial processes usually require exceptionally high titers, yields, and productivity necessitating the engineering of microbes for enhanced fatty acid synthesis (Ledesma-Amaro et al. 2016).

Here, we summarize the current understanding of fatty acid metabolism and highlight key enzymes, including fatty acid synthase, fatty acid desaturases, elongases, and acyltransferases that mainly impact FA composition. We also outline strategies to foster FA and TAG synthesis in both oleaginous and conventional yeasts. Additionally, we discuss successful engineering approaches to alter FA profiles in TAGs and produce length-tailored fatty acids based on practical examples, providing recommendations for host choices to minimize engineering efforts. The review concludes with current challenges, potential solutions, and future prospects for tailoring FA compositions in yeast.

## Fatty acid and triacylglycerol metabolism in yeast

**Fig. 1:**
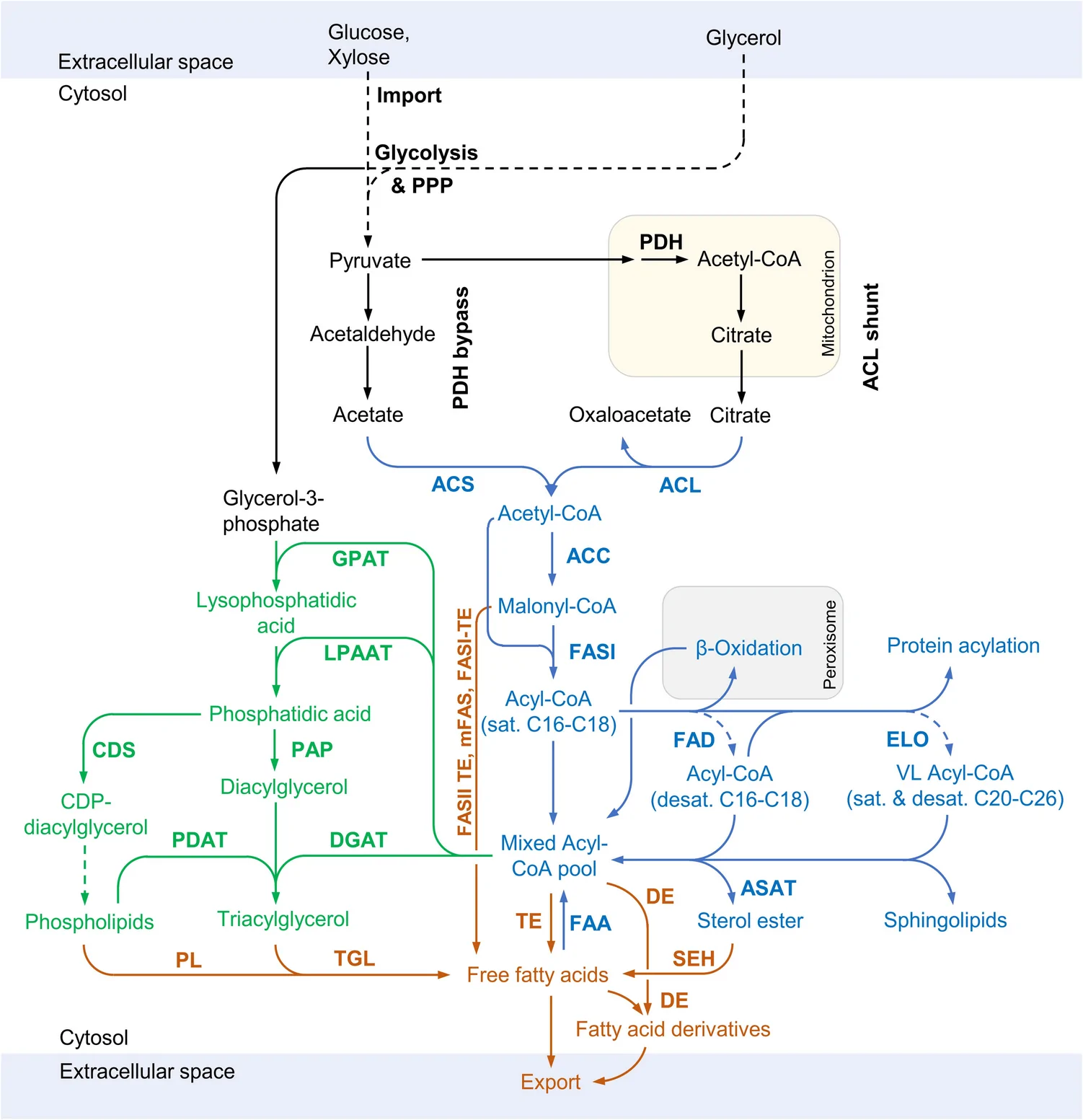
Metabolic pathways for the synthesis of free fatty acids, fatty acid derivatives, and triacylglycerols. Reactions that influence the FA profile of acyl-CoAs (blue), free fatty acids and derivatives (orange), and triacylglycerols and phospholipids (green) are labeled. Multiple reaction steps are denoted by dashed lines. Enzyme/gene abbreviations and precursor pathways are marked in bold. The turnover of acyl groups from acylated proteins and sphingolipids is not shown. Desaturases, elongases, acyltransferases, and lipases are ER or lipid body-associated (enzyme localization is not visualized for these enzymes). PPP, pentose phosphate pathway; PDH, pyruvate dehydrogenase; ACS, acetyl-CoA synthase; ACL, ATP-citrate lyase; ACC, acetyl-CoA carboxylase; FASI fatty acid synthase type I; FASII, fatty acid synthase type II; TE, thioesterase; mFAS, mammalian fatty acid synthase; FASI-TE, fatty acid synthase type I with integrated thioesterase; FAD, fatty acid desaturases; ELO, fatty acid elongases (and auxiliary enzymes); ASAT, Acyl-CoA:sterol acyltransferase; FAA, acyl-CoA synthetase; DE, derivatizing enzymes; SEH, sterol ester hydrolase; TGL, triacylglycerol lipases; PL, phospholipid lipases; GPAT, glycerol- 3-phosphate acyltransferase; LPAAT, lysophosphatidic acid acyltransferase; PAP, phosphatidic acid phosphatase; DGAT, diacylglycerol acyltransferase; CDS, CDP-diglyceride synthetase; PDAT, phospholipid:diacylglycerol acyltransferase

Fermentative FA or TAG production typically relies on inexpensive carbon sources like glucose, xylose, glycerol, or sucrose. To cut costs, side streams from pulp and paper as well as agricultural industries are considered. These carbon sources are taken up by yeast cells and converted to pyruvate via glycolysis or the pentose phosphate pathway. Conventional and oleaginous yeast have adopted distinct strategies for synthesizing the key FA precursor acetyl-CoA from pyruvate within the cytosol, where bulk FA synthesis occurs. Oleaginous yeasts utilize the ATP-citrate lyase pathway (Boulton and Ratledge 1981; Ratledge 2002), while conventional yeasts, such as *S. cerevisiae*, rely on the pyruvate dehydrogenase bypass (Flikweert et al. 1996). In the ATP-citrate lyase pathway, pyruvate is imported into mitochondria and converted to acetyl-CoA, which then reacts with oxaloacetate to form citrate. Citrate is exported from mitochondria and converted back to acetyl-CoA and oxaloacetate by ATP-citrate lyase. Under nitrogen-limited conditions, citrate sequestration through the TCA cycle is inhibited, diverting a larger citrate flux to the cytosol for fatty acid synthesis. Conversely, the pyruvate dehydrogenase bypass in conventional yeasts involves the cytosolic decarboxylation of pyruvate to generate acetaldehyde, followed by conversion to acetate or ethanol, catalyzed by pyruvate decarboxylase, acetaldehyde dehydrogenase, and alcohol dehydrogenase, respectively. Acetyl-CoA synthesis is completed by the activation of acetate with coenzyme A, in an energetically unfavorable reaction catalyzed by acetyl-CoA synthetase, driven by ATP hydrolysis (Fig. 1). FA synthesis is initiated by the conversion of acetyl-CoA to malonyl-CoA by acetyl-CoA carboxylase (Al-Feel et al. 1992). Fatty acid synthase produces long-chain acyl-CoAs by repeated two-carbon extensions of an acetyl moiety consuming malonyl-CoA and NADPH (Wakil and Ganguly 1959). The required reduction equivalent for fatty acid synthesis, NADPH, is produced in the pentose phosphate pathway and to a small degree by malic enzyme (Wasylenko et al. 2015). Long-chain acyl-CoAs then enter pathways for desaturation, elongation, or acyl transfer to form TAGs and phospholipids (PLs), which all compete for FA substrate. Since yeast FAs typically are highly unsaturated, the majority of acyl-CoAs are first subjected to desaturation at the ∆9 position and optionally at the ∆12 and ∆15 positions (Stukey et al. 1989; Zhang et al. 2008). A small fraction of acyl-CoAs are elongated to VLCFAs for sphingolipid synthesis (Oh et al. 1997). Acyl-CoAs of varying chain lengths and degrees of saturation are incorporated into glycerol backbones to form PLs and TAGs, sharing the common precursor phosphatidic acid (PA) (Carman and Henry 1989). In addition, FAs are attached to sterols or proteins to form sterol esters (SE) and acylated proteins for membrane anchoring. TAGs and SE are stored in lipid bodies, which provide FAs for energy production when nutrients become scarce (Seo et al. 2017). TAGs, PLs, and SEs comprise the largest lipid fractions, while acyl-CoAs, FFAs, and sphingolipids are present in smaller quantities (Kaneko et al. 1976). Consequently, PLs, SEs, and particularly TAGs store the bulk of FAs, and their turnover impacts FFA composition (Fig. 1). During starvation, yeast cells mobilize FAs from storage lipids through lipophagic degradation of lipid droplets in the vacuole (Seo et al. 2017). Under these conditions, liberated FAs can be degraded via β-oxidation to generate energy (Lefevre et al. 2013; Weber et al. 2020). Released FFAs are reactivated by acyl-CoA synthetases, and acyl-CoAs are imported into peroxisomes for degradation (Hettema et al. 1996). FFA release from PLs is essential for FA remodeling in PLs to adapt membrane fluidity (Schneiter et al. 1999; Yamada et al. 1977).

The relative activities of the above-mentioned enzymes determine FA compositions of released and stored FAs. Since FA synthesis is an energy-intensive process, the activities of these enzymes are heavily regulated, additionally impacting FA profiles. FA compositions also depend on cultivation conditions, such as temperature, nutrient availability, and stressors as well as on the species and strain selection. The impact of cultivation conditions has been discussed elsewhere (Kolouchová et al. 2015; Sitepu et al. 2013).

### Malonyl-coenzyme A synthesis—acetyl-CoA carboxylase

Malonyl-CoA synthesis in yeast is catalyzed by the cytosolic acetyl-CoA carboxylase (ADP forming acetyl-CoA:carbon-dioxide ligase; Acc1p) from acetyl-CoA and bicarbonate. The reaction is driven by ATP hydrolysis and utilizes biotin as an essential cofactor (Lynen 1967). Malonyl-CoA synthesis is the first committed and major rate-limiting step of de novo fatty acid synthesis and is therefore highly regulated on transcriptional and post-translational levels (Hasslacher et al. 1993; Shi et al. 2014; Woods et al. 1994). Furthermore, malonyl-CoA is essential for the production of very long-chain fatty acids (VLCFAs) catalyzed by ER-resident fatty acid elongases. Acc1p, a 250 kDa/2233 amino acid protein, is comprised of three main domains: biotin carboxylase domain (BC), biotin carboxyl carrier protein domain (BCCP), and carboxyl transferase domain (CT). The Acc1p reaction follows a two-step mechanism that involves the synthesis of a carboxybiotin species in the BC domain, followed by transfer to the CT domain via the BCCP domain and carboxylation of the acetyl moiety to form malonyl-CoA. Acc1p localizes to the cytosol and was reported to associate with mitochondria and microsomal fractions (Ivessa et al. 1997; Sickmann et al. 2003). In mammalian cells and oleaginous yeasts, the presence of the acetyl-CoA precursor citrate stimulates Acc1 activity (and polymerization in mammalian variants) (Botham and Ratledge 1979; Kleinschmidt et al. 1969; Wei and Tong 2018). Besides the cytosolic Acc1p, yeasts also possess a mitochondrial acetyl-CoA carboxylase Hfa1p, which is essential for mitochondrial fatty acid synthesis (Kearsey 1993).

### Regulation of acetyl-CoA carboxylase

Transcriptional control of *ACC1* and expression of genes encoding other major enzymes in FA metabolism have been reviewed by others (Santomartino et al. 2017; Tehlivets et al. 2007) and will not be discussed here. On the protein level, Acc1p activity is regulated through post-translational modification (phosphorylation), allosteric control, and the degree of biotinylation. Acc1p is phosphorylated by the AMP-dependent kinase Snf1p, which is activated at high AMP/ATP ratios. Snf1p phosphorylates Acc1p at serine 1157 and likely serine 659 (recognition sites Φ-X-R-XX-S-XXX-Φ). Mutating these residues in *ACC1* leads to a considerable increase in Acc1p activity, corresponding to that of a *snf1*Δ mutant, in which Acc1p is deregulated (Shi et al. 2014). Additionally, Acc1p activity is regulated through feedback inhibition via its product malonyl-CoA and via the end products of fatty acid synthesis, long-chain acyl-CoAs, as first reported for chicken Acc1p (Goodridge 1972; Shirra et al. 2001).

### Fatty acid chain length control by acetyl-CoA carboxylase

Acc1p activity indirectly influences the spectrum of synthesized acyl-CoAs by altering the relative concentrations of acetyl-CoA and malonyl-CoA (Sumper et al. 1969). In fungal FAS, the malonyl/palmitoyl transferase (MPT) domain is crucial for loading malonyl onto the acyl carrier protein domain (ACP) and terminating finished acyl chains by transferring them to CoA, with both substrates competing for the active site. At elevated malonyl-CoA concentrations, the loading activity predominates over termination, leading to an increased relative abundance of C18 acyl-CoA products (Kawaguchi et al. 1980). Conversely, at low malonyl-CoA/acetyl-CoA ratios, the termination activity is enhanced, resulting in the elevated synthesis of C14 and C16 species. Thus, the relative rates of acetyl-CoA and malonyl-CoA synthesis impact the spectrum of synthesized long-chain acyl-CoAs.

### Fatty acid synthase

In *S. cerevisiae*, bulk fatty acid synthesis is catalyzed by the cytosolic type I fatty acid synthase (FAS), which is composed of two polypeptide chains Fas1p (beta subunit) and Fas2p (alpha subunit), encoded by *FAS1* and *FAS2*, respectively. The subunits assemble into a 2.6-MDa heterododecamer with a barrel-shaped structure (Jenni et al. 2006). Each protomer harbors multiple domains: Fas1p contains acetyltransferase (AT), enoylreductase (ER), dehydratase (DH), and malonyl/palmitoyl transferase (MPT) domains, while Fas2p contains MPT (second part), acyl carrier protein (ACP), ketoreductase (KR), ketosynthase (KS), and phosphopantetheine transferase (PPT) domains. Notably, the domain organization can slightly differ in other yeast species. For instance, in *R. toruloides*, the Fas2p contains two ACP domains (Zhu et al. 2017). The spherical FAS complex is composed of two chambers separated by a central disc. Each chamber harbors all catalytic domains thrice, forming three reaction centers, wherein ACP domains attached on flexible tethers shuttle fatty acid intermediates and acetyl and malonyl moieties between active sites (Jenni et al. 2006). The 4′-phosphopantetheine prosthetic group is transferred to the ACP domain by an intrinsic PPT activity (PPT domain) (Fichtlscherer et al. 2000). The tight organization promotes high intermediate concentrations and thus facilitates a high synthesis rate.

FA synthesis follows a multistep process that involves priming, repeated elongation, and termination. Fatty acid synthesis is initiated through the loading of an acetyl or acyl moiety via the AT acetyl/acyltransferase domain onto ACP, followed by its transfer into the KS domain, functioning as a priming unit for FA synthesis. Subsequently, a malonyl unit provided by the MPT domain is likewise shuttled via ACP to the KS domain, remaining attached to ACP. Subsequent decarboxylation of the malonyl moiety presumably leads to an enolate-driven condensation with the acetyl residue, generating a ketoacyl intermediate (Arnstadt et al. 1975; Heath and Rock 2002). The ACP tethered intermediate is further transferred to the KR, DH, and ER domains for NADPH-dependent keto reduction, dehydratization, and NADPH-dependent enoyl-reduction, respectively, yielding a saturated acyl chain that is extended by two carbon atoms. The extension proceeds until a final chain length of typically 16–18 carbon atoms is reached. Chain termination is facilitated by the MPT, transferring the fatty acid from ACP onto CoA (Lynen 1961; Sumper et al. 1969).

Interestingly, the structural organization and mechanisms of chain termination differ among kingdoms of life, even though the chemistry of fatty acid synthesis is conserved (reviewed in Heil et al. (2019)). In mammals, FAS assembles into a flexible X-shaped (“gingerbread man”-like) homodimer. Each protomer contains all catalytic domains on a single polypeptide chain, except for the PPT domain, which is expressed as a separate protein (Maier et al. 2008). Substrate shuttling is facilitated by swinging and swiveling of the leg structure and rearrangement of domains in the arms to connect the ACP with the catalytic domains (Brignole et al. 2009). Chain termination is carried out by a thioesterase domain, which cleaves the thioester bond at specific acyl chain lengths (typically C16 in human FAS), determining the produced FA spectrum (Chakravarty et al. 2004). In prokaryotes, fungal and animal mitochondria, and plants, FAs are produced by disassociated type II FAS systems. Here, all domains are expressed as individual proteins, and ACPs transfer intermediates between the catalytic domains by diffusion (Beld et al. 2015). In prokaryotes, finished acyl chains are directly transferred from ACPs to acyltransferases for lipid synthesis, while in plant plastids, fatty acid synthesis is terminated by length-specific thioesterases that release free fatty acids (FFA). Notably, bacteria of the *Corynebacterineae* group (*Corynebacteria*, *Mycobacteria*, and *Nocardia*—CMN bacteria) possess a type I fungal-like FAS, which harbors all domains but the PPT domain on a single polypeptide (Boehringer et al. 2013). The fungal, mitochondrial type II FAS displays a bimodal product spectrum, synthesizing C8 and C16–C18 fatty acids. Octanoic acid production is presumably required for lipoic acid synthesis (Brody et al. 1997), a crucial cofactor in decarboxylation reactions (Schonauer et al. 2009). Since bacterial, yeast, and animal FAS systems differ in their mechanisms for chain termination, heterologous expression and engineering of chain length-determining domains offer the opportunity for modifying FA profiles in yeast.

### Chain length control in the fungal fatty acid synthase

The mechanism that defines the final FA chain length in the FAS complex involves a competition between repeated chain extension through condensation and termination from the synthase complex by transferring it to CoA (or releasing the FFAs in mammals). Thus, both the KS and MPT domains exert chain length regulatory functions. The MPT domain exhibits comparable transferase (termination) rates for unsaturated acyl substrates with chain lengths ranging from 6 to 18 carbon atoms, while shorter acyl substrates are transferred at lower rates (Schweizer et al. 1970). Furthermore, as previously discussed, the termination rate mediated by the MPT domain is modulated by malonyl-CoA concentrations. The KS domain, on the other hand, controls acyl chain length through restricted space in the acyl-binding tunnel (Gajewski et al. 2017; Johansson et al. 2008), which prevents the extension of C18 chains and promotes the release of long-chain species. Additionally, the acetyltransferase (AT) domain's priming activity influences chain length to a lesser degree, as increased AT activity can lead to earlier termination of acyl chains. The interplay of these functions defines the FA chain length profile of the fungal FAS, resulting in C14–C18 product spectra (Sumper et al. 1969). The AT domain can load alternative priming units, such as propionyl-CoA or short-branched or functionalized acyl-CoA units. This allows FAS to produce odd-chain, branched, or functionalized fatty acids, albeit at reduced efficiencies. Priming with shorter-chain acyl moieties typically results in their extension to 16–18 carbon atoms (Pirson et al. 1973), which is unfavorable when aiming to produce short- or medium-chain FAs. Therefore, preventing the activation of shorter FAs, which is required for acyl loading, or engineering the AT domain may help circumvent their extension. Further details on chain length regulation with detailed mechanistic insights are summarized by Heil et al. (2019).

### Fatty acid synthase—regulation on protein level

FAS activity is regulated by feedback inhibition from its final products, palmitoyl-CoA and stearoyl-CoA. These molecules remain attached to the synthase complex in vitro, thereby interfering with its termination function (Sumper and Träuble 1973). Additionally, FAS is subject to phosphorylation at various sites. Notably, mutations of Fas2p phosphorylation sites (S1440, S1640, and S1827) to glutamate resulted in increased production of C18 fatty acids and decreased production of C16 fatty acids, along with reduced cell growth (Martínez-Montañés et al. 2020). A recent discovery identified Tma17p, a regulatory subunit of the yeast FAS referred to as the γ subunit, which modulates FAS activity based on the availability of the cofactor NADPH. Tma17p also prevents the synthesis of futile reactive oxygen species in the ER domain (Singh et al. 2020).

### Fatty acid desaturation

Fatty acid desaturases introduce double bonds into saturated and unsaturated fatty acids to maintain plasma membrane fluidity under changing environmental conditions (Los and Murata 1998). Most FA double bonds in living organisms exert cis configuration, which effectively interferes with the tight packing of acyl tails in membranes, thus controlling membrane fluidity. The desaturation reaction requires molecular oxygen, a di-iron cofactor coordinated by conserved histidine motifs, and reduction equivalents provided by cytochrome b5 in yeasts and animals or ferredoxin in bacteria. Cytochrome b5 and ferredoxin are initially reduced in an NADH-dependent reaction by cytochrome b5 reductases or ferredoxin reductases, respectively (Stukey et al. 1990; Wada et al. 1993). FAs targeted by desaturases may be linked to CoA and ACP or be part of phospholipids. In animals and fungi, desaturases are ER membrane-associated and utilize fatty acyl-CoAs and phospholipids, while in plants (mostly in plastids) and some bacteria, soluble fatty acyl-ACP or membrane-bound acyl-glycerolipid desaturases are found (Los and Murata 1998). Most prokaryotes introduce double bonds anaerobically during fatty acid synthesis. Fatty acid desaturases are, moreover, classified based on their regioselectivity into three groups: saturated fatty acyl-CoA ∆9 desaturases or SCDs (stearyl-CoA desaturase), omega desaturases, and front-end desaturases (Hashimoto et al. 2008). SCDs introduce the first double bond in saturated acyl-CoAs with chain lengths of 14 to 19 carbon atoms (mostly C16 and C18) in the ∆9 position. Omega desaturases sequentially introduce additional double bonds between the initial double bond and the omega terminus, i.e., first at the Δ12 position, then at the Δ15 position. Front-end desaturases catalyze double bond formation at positions ∆4, ∆5, ∆6, and ∆8 between the carboxy moiety and an existing central double bond (Hashimoto et al. 2008). Despite high similarity among protein sequences of regioselectivity classes, distinctions can be made based on differences in the consensus sequences of the conserved histidine motifs (Hashimoto et al. 2008). Since animal and fungal desaturases are transmembrane proteins, only very few structures have been unraveled, whereas the structures of soluble plastidial desaturase are well characterized (Nachtschatt et al. 2020). Fungal ∆9-desaturases display high homology to their mammalian counterparts (Bai et al. 2015; Wang et al. 2015), exhibiting a mushroom-like structure that typically features two transmembrane domains spanning the ER membrane (Stukey et al. 1990). The active site is located on the cytosolic side within the “mushroom cap” structure, which indicates that acyl-CoAs are directly sequestrated from the cytosol or ER membrane. Additionally, the fungal SCDs possess a C-terminal cytochrome b5 domain, though yeasts also express independent cytochrome b5 proteins (Mitchell and Martin 1995). In yeast, SCDs are essential and sufficient to regulate membrane fluidity, but various yeast species additionally express ∆12- and ∆15-desaturases (Kainou et al. 2006; Zhang et al. 2008). Front-end desaturases have not been identified in yeast but are present in other fungi. *S. cerevisiae* only harbors a sole desaturase, Ole1p (9-desaturase), and is therefore unable to synthesize polyunsaturated fatty acids (Stukey et al. 1989). It was reported that overexpression of mammalian SCDs in yeast led to the incorporation of zinc ions instead of iron, rendering the enzymes inactive (Bai et al. 2015; Wang et al. 2015).

### Regulation of fatty acid desaturases

In *S. cerevisiae*, *OLE1* expression is induced by hypoxia, reduced temperatures, and the presence of saturated FAs, while unsaturated FAs lead to its repression (Bossie and Martin 1989; Kwast et al. 1999; Nakagawa et al. 2002). Similar regulatory effects have been observed in other yeasts (De Angelis et al. 2016; Lu et al. 2000; Yu et al. 2012a, b). *OLE1* expression and mRNA stability in *S. cerevisiae* are regulated by the homologous transcription factors Mga2p and Spt23p, which are anchored in the ER membrane and subject to proteasomal cleavage depending on ER membrane fluidity. After cleavage, the regulatory domain migrates to the nucleus to control *OLE1* transcription (Hoppe et al. 2000; Kandasamy et al. 2004). Homologues of Mga2/Spt23 have been identified in other yeast species (Burr et al. 2016; Liu et al. 2015; Micolonghi et al. 2012; Yu et al. 2012a, b), and the respective null mutants show reduced growth and altered fatty acid profiles (Burr et al. 2016; Micolonghi et al. 2012; Yu et al. 2012a, b). Thus, changes in *MGA2* expression can be exploited to render fatty acid profiles in yeast (Zhang et al. 2022a, b).

### Fatty acid elongases

Fatty acid elongases and their auxiliary enzymes are ER-resident integral transmembrane proteins that extend medium- or long-chain fatty acyl-CoAs (C12–C18) to very long-chain acyl-CoAs (C22–C26 or longer). Their reaction mechanism is reminiscent of fatty acid synthesis utilizing malonyl-CoA and NADPH in subsequent steps. However, in contrast to bulk FA synthesis, the extended acyl chains remain CoA-bound rather than being attached to ACP (Nie et al. 2021; Oh et al. 1997). Based on their selectivity, FA elongases can be classified into elongases that convert saturated or monounsaturated fatty acids and elongases that convert polyunsaturated fatty acids (Hashimoto et al. 2008). The former class, present in fungi, displays a large variety in substrate acyl chain length. For instance, the three homologues found in *S. cerevisiae*—Elo1p, Elo2p, and Elo3p—convert C13/C14–C16 (Toke and Martin 1996), C16–C24, and C16–C26 (Denic and Weissman 2007; Oh et al. 1997), respectively. The second elongase class, active on PUFAs, is found in animals, fungi, protists, and others (Hashimoto et al. 2008; Xue et al. 2013). Fatty acid elongases only catalyze the rate-limiting condensation reaction, while the microsomal proteins Ybr159wp (Ifa38p), Phs1p, and Tsc13p catalyze the subsequent steps of keto reduction, water abstraction, and enoyl-reduction, respectively (Denic and Weissman 2007; Han et al. 2002; Kohlwein et al. 2001). Due to their overall hydrophobic nature, few structures of fatty acid elongases and auxiliary enzymes have been resolved. Topological analysis indicates the presence of multiple transmembrane helices (Stukey et al. 1990). Insights into elongase structures can be gained from the human homologue Elovl7p, which displays 45–50% similarity to the yeast variants (Nie et al. 2021). Elovl7p forms an inverted barrel structure from six transmembrane helices, with an adjacent seventh transmembrane helix embedded in the ER membrane. A narrow substrate binding tunnel protrudes from a small opening on the cytosolic side towards the closed end. Structural investigation indicated that acyl-CoA substrates and ketoacyl-CoA products enter and leave the active site between two transmembrane helices along the plane of the ER membrane. Spatial constraints in the acyl chain binding pocket define the final chain lengths of synthesized products, as confirmed by mutational analysis of residues at the bottom of the tunnel (Denic and Weissman 2007). A conserved HXXHH histidine motif lies in the active site, essential for the elongation reactions (Nie et al. 2021). Since VLCFAs are involved in various cellular functions, including protein trafficking, stabilizing the nuclear envelope curvature, and synthesis of sphingolipid (Denic and Weissman 2007), elongase mutants exhibit restricted or impaired growth. In *S. cerevisiae*, Elo1p and FAS have partially overlapping functions in extending C14 to C16/C18. Moreover, Elo2p and Elo3p both elongate C16 to C22 or C26 (Rössler et al. 2003). Elongase genes are repressed in the stationary phase or during nitrogen limitation, indicating that elongase regulation is growth-associated (Gasch et al. 2000). The activities of fatty acid elongases are regulated through (de)phosphorylation, depending on plasma membrane sphingolipid levels (Olson et al. 2015; Zimmermann et al. 2013).

### Fatty acid uptake, transport, and export

FA uptake in yeast is mediated by the Fat1p membrane protein, which exhibits long-chain FA transport and VLCFA CoA synthetase activity (Zou et al. 2002). During transport, Fat1p interacts with fatty acyl-CoA synthetases Faa1p and Faa4p for concatenated fatty acid import and activation to acyl-CoAs (Obermeyer et al. 2007), a process known as vectorial acylation that enables efficient FA trapping and metabolic utilization. Endocytosis significantly contributes to FA uptake, as it was shown that deletions of Ypk1, a protein kinase involved in the regulation of endocytosis, or other associated proteins can reduce FA uptake by up to 50% (Jacquier and Schneiter 2010). Acyl-CoA synthetases also play a crucial role in converting FFAs from lipid turnover into their activated acyl-CoA form in an ATP-dependent reaction. *S. cerevisiae* possesses five homologues (Faa1p to Faa4p and Fat1p), of which Faa1p and Faa4p contribute major activities, both preferentially converting long-chain substrates (Faergeman et al. 2001; Knoll et al. 1994). Faa2p is involved in peroxisomal FA activation, while the function of Faa3p remains unknown. Notably, Fat1p not only exhibits VLCFA-CoA synthetase activity, but also activates long acyl chain substrates (Scharnewski et al. 2008). Triple deletion strain *fat1*Δ *faa1*Δ *faa4*Δ cannot metabolize supplemented FFAs, though FFAs are still imported into cells when other carbon sources are depleted (Scharnewski et al. 2008). In *Y. lipolytica*, FA uptake differs from *S. cerevisiae*, as Fat1p does not participate in FA uptake and Faa1p is not required for FA metabolism, suggesting that FFAs are directly activated in peroxisomes (Dulermo et al. 2015, 2014).

The transport of cytosolic acyl-CoAs is facilitated by the dedicated transport protein Acb1p. Furthermore, it was suggested that acyl-CoAs are transferred through diffusion or by protein-membrane or protein–protein contact (transfer to ER membrane or desaturases). In vitro experiments with purified FAS could not confirm transport through diffusion, but hinted towards protein–protein/membrane contact exchange (Sumper and Träuble 1973). Interestingly, when *ACB1* is deleted, cells are viable (Schjerling et al. 1996) and initially propagate slowly, but revert to a rapid growth phenotype (Gaigg et al. 2001), consolidating the hypothesis of alternative transport mechanisms. It was shown that Acb1p aids in terminating acyl-CoAs from the FAS complex and thereby influences intrinsic acyl-CoA compositions. Furthermore, Acb1p is pivotal for VLCFA synthesis and membrane trafficking. Under nutrient-depleted conditions, FAs in *S. cerevisiae* are mobilized from lipid droplets and transferred to peroxisomes for degradation. Under these conditions, lipid bodies, peroxisomes, and mitochondria come into close contact, forming organelle contact sites to promote rapid metabolite exchange (Binns et al. 2006; Shai et al. 2018). Arf1p, a GTPase protein, plays a pivotal role in the generation of organelle interaction sites, and strains expressing a hyperactive Arf1 mutant display enlarged lipid bodies and reduced β-oxidation activity (Enkler et al. 2023). Long-chain acyl-CoA import into peroxisomes is mediated by transport proteins Pxa1p and Pxa2p. During transport, CoA is released on the cytosolic side, while FAs are transferred into the peroxisome, activated by Faa2p and Fat1p, and subsequently degraded (van Roermund et al. 2012).

In *S. cerevisiae faa1Δ faa4Δ* strains, released FFAs stemming from lipid turnover cannot be reactivated and are therefore exported from cells to prevent toxicity (Scharnewski et al. 2008). Although exporting fatty acids is not typical for most microorganisms due to significant energy loss, it becomes essential for survival in strains with impaired acyl-CoA synthetase activity (Darwiche et al. 2017). FFA export phenomena are still mostly obscure, though certain proteins have been identified that contribute to this process. The multi-drug resistance membrane protein, Tpo1p, exports polyamines and medium-chain fatty acids (Legras et al. 2010). Moreover, the yeast Pry1p to Pry3p proteins are involved in FFA export, which display partially redundant functions, and the presence of a single homologue is essential for the survival of *faa1*Δ *faa4*Δ strains (Darwiche et al. 2017). The secretion of FFAs and FA derivatives facilitates efficient downstream processing, which is further discussed in later sections. It is likely that the chain length selectivity of FA transporting and activating enzymes influences FA profiles in yeast.

### β-Oxidation

β-Oxidation proceeds in a cyclic fashion, resembling a reversed FA synthesis. The first committed and rate-limiting step is catalyzed by Pox1p, the fatty acyl-CoA oxidase, which requires molecular oxygen and generates hydrogen peroxide. The deletion of *POX1* results in impaired β-oxidation activity (Dmochowska et al. 1990). Subsequent steps in β-oxidation are carried out by the bifunctional enzyme Fox2p, which has trans- 2-enoyl-CoA hydratase and 3-hydroxy-acyl-CoA dehydrogenase activities, and by Pot1p, which acts as a 3-oxo-acyl-CoA thiolase (Hiltunen et al. 1992; Igual et al. 1991). Each cycle shortens the FA chain by two carbon atoms, producing one molecule of each acetyl-CoA, NADH, and hydrogen peroxide. Auxiliary enzymes assist in degrading unsaturated fatty acids with specific doublebond configurations that cannot be processed by the main enzymes (Gurvitz et al. 1998). While *S. cerevisiae* possesses a single POX gene with a broad substrate spectrum, *Y. lipolytica* harbors five *POX* genes with different chain length specificities (Wang et al. 1999). β-Oxidation can be exploited to produce truncated fatty acids and β-oxidation intermediates by interfering in the degradation process, such as through the deletion of short-chain acyl-CoA oxidase or by disrupting further steps in the β-oxidation cycle (Groguenin et al. 2004). The deletion of Pot1 for instance promotes methyl ketone synthesis, and the expression of intermediate-specific thioesterases further facilitates intermediate release (Hanko et al. 2018). Additionally, the chain-shortening process provides an opportunity to shift existing double bonds towards the carboxy side of the fatty acid, which is relevant in the synthesis of docosahexaenoic acid in mammals (Hashimoto et al. 2008). In the presence of glucose, β-oxidations genes are repressed, while the availability of oleic acid in the absence of glucose leads to strong induction of β-oxidation genes and peroxisome proliferation (Dmochowska et al. 1990).

### Triacylglycerol, phospholipid, and sterol ester synthesis and degradation

TAGs and PLs are synthesized from the common precursor phosphatidic acid (PA). The first step in PA synthesis is the acyl-CoA-dependent incorporation of an FA at the sn- 1 position of glycerol- 3-phosphate or dihydroxy acetone phosphate (DHAP), producing lysophosphatidic acid or acyl dihydroxy acetone phosphate, respectively. In *S. cerevisiae*, this reaction is catalyzed by glycerol- 3-phosphate acyltransferases Sct1p (Gat2p) and Gpt2p (Gat1p). Gpt2p equally accepts G3P and DHAP as substrate, whereas Sct1p prefers G3P over DHAP. Furthermore, Gpt2p efficiently incorporates most long-chain FAs (C16:0, C16:1, C18:1), but has slightly lower activity for C18:0. Sct1p, on the other hand, shows higher efficiency for incorporating palmitoyl-CoA (Zheng and Zou 2001). Overexpression of *SCT1* leads to a relative increase of C16:0 stored in TAGs and PLs, while its deletion results in a reduction of C16:0. The deletion of *GPT1* has little impact on the FA profiles in total lipids (De Smet et al. 2012). While strains with single deletions of *SCT1* or *GPT2* display wild-type-like growth, double deletions are synthetically lethal (Zheng and Zou 2001). Ayr1p (1-acyl dihydroxyacetone phosphate reductase) reduces 1-acyl DHAP to lysophosphatidic acid, allowing for further metabolization (Athenstaedt and Daum 2000). In the second step of PA synthesis, another acyl chain is transferred from acyl-CoAs into the sn- 2 position of LPA. This reaction is primarily catalyzed by Slc1p and Ale1p (also known as Slc4p or Lpt1p), as evidenced by the lethality of their double deletions (Benghezal et al. 2007; Nagiec et al. 1993). Besides their activity for LPA, Slc1p and Ale1p catalyze the acyl transfer to other lysophospholipids (Tamaki et al. 2007). Investigations of fatty acid profiles in PLs in *slc1*∆ and *ale1*∆ strains revealed that Slc1p preferentially incorporates C18:1, and is also responsible for medium-chain fatty acid transfer (C14–C10) (Shui et al. 2010). In comparison, Ale1p accepts a broad spectrum of long-chain saturated and unsaturated acyl-CoA substrates (Benghezal et al. 2007; Chen et al. 2007; Tamaki et al. 2007).

PL synthesis starts with cytidylyltransfer to PA catalyzed by Cds1p to produce CDP-DAG (Shen et al. 1996), while TAG synthesis is initiated by dephosphorylation of PA to diacylglycerol (DAG), catalyzed by phosphatidate phosphatases Pah1p, Lpp1p, Dpp1p, and App1p (Chae et al. 2012; Han et al. 2006; Toke et al. 1998; Wu et al. 1996) and subsequent esterification of a third acyl chain. Acyl chain transfer to DAG is conferred by Lro1p (Phospholipid acyltransferase) and Dga1p, with Lro1p being primarily active during exponential growth and Dga1p contributing most DAG acyl transfer activity during the stationary phase (Oelkers et al. 2002). Dga1p utilizes acyl-CoAs as an acyl donor, whereas Lro1p transfers acyl chains from the sn- 2 position of phospholipids to DAG. In vitro experiments revealed that Dga1p preferentially incorporates C16:0 and C18:1, but is also active on C14:0, C18:0, C18:2, and C20:4 (Oelkers et al. 2002). Lro1p’s acyl substrate preferences are mostly unknown, but it was shown that unsaturated acyl donors are preferred over saturated ones: i.e., PLs with 18:1/18:0 are preferred over 18:0/18:0 (Feng et al. 2019). TAG synthesis is primarily controlled by the activity of phosphatidate phosphatase Pah1p, which is strictly regulated through phosphorylation, localization (ER attachment or cytosolic localization), and the abundance of nucleotides and phospholipids (Karanasios et al. 2010; Wu and Carman 1996, 1994). Sterol esters are synthesized by the ER-resident enzymes Are1p and Are2p from various sterol species and acyl-CoAs. Both Are1p and Are2p predominantly utilize C16:1-CoA and C18:1-CoA as acyl donor substrates, while saturated long-chain acyl-CoAs are incorporated at lower rates (Zweytick et al. 2000). Are1p and Are2p possess minor acyl-CoA-dependent TAG synthase activities (Sandager et al. 2002). Their transcription depends on heme and oxygen availability (Valachovič et al. 2002).

TAG lipases localize to lipid bodies and play a crucial role in releasing FFAs from TAGs, PLs, SEs, and intermediates. This process is essential for fatty acid remodeling in PLs, provides FAs for energy production, and supplies FAs and DAGs for the synthesis of new PLs (Rajakumari et al. 2010; Schneiter et al. 1999). *S. cerevisiae* possesses five TAG lipase homologues, encoded by *TGL1*-*TGL5*, with Tgl3p and Tgl4p contributing major lipase activities (Athenstaedt and Daum 2005, 2003). Tgl3p does not show a preference for specific acyl chain lengths, while Tgl4p exhibits the highest activity for TAGs containing myristic and palmitic acids, and Tgl5p prefers TAGs with very long-chain fatty acids (VLCFAs, C26:0) (Athenstaedt and Daum 2005). Tgl1p has sterol ester hydrolysis activity alongside Yeh1p and Yeh2p (Köffel et al. 2005), while Tgl2p appears to be involved in mitochondrial lipid metabolism (Ham et al. 2010). Tgl3p, Tgl4p, and Tgl5p also function as acyltransferases for lysoPLs (Rajakumari and Daum 2010a, b). Specifically, Tgl3p’s acyltransferase activity and Tgl4p’s lipase activity are essential for sporulation and cell cycle progression, respectively (Kurat et al. 2009; Rajakumari and Daum 2010a). Yju3p has been identified as the major monoacylglycerol lipase (Heier et al. 2010). TGLs are strongly activated when cells enter vegetative growth to mobilize DAGs and FAs for membrane propagation (Kurat et al. 2006). Tgl4p is activated in dependence on the cell cycle stage, initially activated in the G1/S transition through phosphorylation by the cyclin-dependent kinase 1 Cdc28p to provide lipids for the daughter cell (Kurat et al. 2009). The expression of *TGL3* is strongly induced when stationary phase cells are transferred to a fresh medium (Lu et al. 2019), yet the regulatory mechanisms at the protein level for Tgl3p remain unclear. In *Y. lipolytica*, mostly extracellular lipases have been extensively studied due to their industrial relevance. However, intrinsic TAG lipases, namely Tgl3p and Tgl4p, have also been identified. Tgl3p appears to activate Tgl4p, and the knockout of either protein inhibits TAG mobilization (Dulermo et al. 2013). It is worth noting that lipase acyl-length preferences can be modified by engineering residues in the active site to accommodate specific acyl substrates (Schmitt et al. 2002). Lastly, phospholipases are responsible for releasing FFAs from various PL species by cleaving acyl chains at the sn- 1 and/or sn- 2 positions (Lee et al. 1994; Merkel et al. 1999). FA remodeling in PLs is further facilitated by acyl-CoA or PL-dependent acyltransferases, which has been reviewed by Patton-Vogt and de Kroon (2020).

## Engineering fatty acid metabolism

Commercial processes for biotechnological production require high yields, titers, and volumetric productivities, which wild-type organisms often fail to meet. Therefore, metabolic engineering is employed not only to alter the FA profile for specific products but also to trim the strains towards industrial fitness. Common strategies for yeast metabolic engineering include push, pull, block engineering, and deregulation of key enzymes in the targeted metabolic pathways. The following chapter summarizes these strategies to improve the production of FFAs, FA derivatives, and TAGs and discusses factors that influence the fatty acid profile.

### Enhancing terminal reactions—pull engineering

FA overproduction can be achieved by either secreting FFAs into the cultivation medium or storing FAs in their esterified form in TAGs. In *S. cerevisiae*, FFA secretion requires the inactivation of acyl-CoA synthetases Faa1p and Faa4p (Scharnewski et al. 2008). When *FAA* genes are deleted, FFAs are continuously released from TAGs, SE, and PLs through the action of various lipases. Due to the lack of *FAA* activity, FFAs cannot re-enter the metabolism and are exported from cells, presumably to prevent FA-induced toxicity. FA secretion facilitates rapid and cost-effective downstream processing, eliminating the need for cell disruption, purification, and transesterification/saponification of fatty acids. Deletions of acyl-CoA synthetases have been successfully implemented in various yeast species including *S. cerevisiae*, *Y. lipolytica*, *Pichia pastoris*, and *Ogataea augusta*, promoting the secretion of 0.5–2 g/L of FFAs in shake flask cultivations with minimal medium (Cai et al. 2022; Gao et al. 2022; Kobalter et al. 2023; Ledesma-Amaro et al. 2016; Yu et al. 2018; Zhou et al. 2016). Nevertheless, a significant fraction of FAs remains stored as TAGs and SEs within the cells, indicating further potential for FA secretion (Ledesma-Amaro et al. 2016; Yu et al. 2018).

FFA secretion in *FAA*-deficient strains can be enhanced by expressing heterologous or endogenous thioesterases that cleave the thioester bonds of acyl-CoA and release FFAs. This strategy has been successfully employed in various yeast species. For instance, the overexpression of an N-terminally truncated thioesterase from *E. coli* (‘*TesA*), in an acyl-CoA synthetase-deleted *Y. lipolytica* strain, increased secreted FA titers from 0.7 to 2.3 g/L (Ghogare et al. 2020). Similarly, overexpressing the truncated *E. coli* thioesterase in an engineered *P. pastoris* strain led to a 40% increase in FA secretion (Kobalter et al. 2023). Overexpression of endogenous peroxisomal thioesterases or plant thioesterases has also been shown to improve FFA production in *S. cerevisiae* (Li et al. 2014). *E. coli TesA* is the most commonly employed thioesterase due to its high activity on various saturated and unsaturated long-chain acyl-CoA substrates (highest for C14–C18) (Bonner and Bloch 1972) and acyl-ACP substrates. Recently, a *TesA* variant (R64 C) with twofold improved catalytic activity has been identified (Shin et al. 2016).

Heterologous thioesterase expression not only improves FFA titers and productivity, but also offers opportunities for fatty acid profile engineering, as certain variants are highly specific towards chain lengths and degrees of desaturation (Jing et al. 2011). Jing et al. investigated the substrate specificities of 31 different acyl-ACP thioesterases, which are directly compatible with type II FAS systems or may be incorporated into type I FAS systems. In comparison, acyl-CoA thioesterases release FAs directly from the acyl-CoA pool (Kirkby et al. 2010), enabling tailored FFA production without incorporating them into the yeast FAS complex. Consequently, thioesterases have been employed for the production of various fatty acid products with tailored chain lengths (i.e., medium-chain fatty acids, see product section).

Besides using thioesterase, flux towards FFAs can be further enhanced by overexpressing diacylglycerol acyltransferase *DGA1* and the major TAG lipase *TGL3*. This approach led to a 1.7-fold increase in FFA secretion in a *S. cerevisiae* strain with impeded FFA activation and β-oxidation (Leber et al. 2015). Furthermore, the implementation of this strategy in *P. pastoris* resulted in a 42% improvement in FA secretion (Kobalter et al. 2023). Alternatively, Ferreira et al. blocked TAG synthesis and diverted FA flux through phospholipids, facilitating enhanced FFA secretion with phospholipases (Ferreira et al. 2018). The resulting strain not only showed enhanced FA production, but also displayed eightfold increased PL concentrations. Consequently, this approach could be employed for PL overproduction. Interestingly, overexpressing phospholipases did not significantly improve FFA production, suggesting that phospholipase activity was not limiting in this study.

Another effective strategy for FFA production involved timed uncoupling of TAG synthesis and FFA secretion through induced expression of TAG lipases in later stages of the fermentation. In this way, large amounts of TAGs are initially accumulated, followed by efficient FFA release. This is particularly attractive as TAG production is more efficient than continuous FFA secretion in yeast. So far, this strategy has only been implemented in the oleaginous bacterium *Rhodococcus opacus*, yielding the highest FFA titer reported in literature at 50.2 g/L (Kim et al. 2019).

Aside from FFA production, efficient TAG accumulation is conferred by overexpressing Dga1 and Acc1, which catalyze the major rate-limiting steps in TAG synthesis (Tai and Stephanopoulos 2013). In *Y. lipolytica*, overexpression of *DGA1* and *ACC1* from an engineered *TEF1* promoter increased the lipid content from roughly 10% in the wild-type strain up to 62% in the modified strain (Tai and Stephanopoulos 2013). In another study, high TAG-accumulating *S. cerevisiae* strains were crossed, and among other modifications, a mutated *ACC1* variant and *DGA1* were overexpressed in the segregants. The best combinatorial strain accumulated up to 65% neutral lipids, representing the highest lipid content achieved in *S. cerevisiae* to date (Arhar et al. 2021). Notably, various oleaginous yeast species accumulate lipid contents of 20–65% under nitrogen-limited conditions without overexpressing *DGA1* and *ACC1* (Abeln and Chuck 2021).

### Interference with competing pathways—block engineering

Carbon flux can be directed into target pathways by deleting or downregulating competing reactions that consume precursors, intermediates, or desired products. For FA production, research has focused on disrupting or downregulating β-oxidation, neutral lipid storage, glycogen synthesis, trehalose synthesis, and the release of fermentation products. β-Oxidation is inhibited by deleting *POX1* (fatty acid oxidase), *PXA2/PXA1* (peroxisomal transport proteins), and *FAA2/FAT1* (acyl-CoA synthetases), or combinations thereof. The literature presents mixed results regarding the effectiveness of this strategy for FA overproduction, as demonstrated in several studies with the yeast *S. cerevisiae*. Leber et al. reported a 2.7-fold increase in extracellular FFAs by deleting *POX1*, *PXA2*, and *FAA2* in an *faa1*Δ *faa4*Δ *fat1*Δ background (Leber et al. 2015). Furthermore, Li et al. have also reported an improvement in FA titer of 31% by deleting *POX1* in an *faa1*Δ *faa4*Δ strain (Li et al. 2014). In contrast, some studies indicated that deletions of genes involved in β-oxidation could not increase FA production (Cai et al. 2022) or even lead to a reduction in titers (Runguphan and Keasling 2014). Leber et al. pointed out that a combination of *pox1*, *pxa2*, and *faa2* deletions is required to fully interfere with β-oxidation.

Another strategy to alleviate competing pathways for FA production focuses on the disruption of neutral lipid storage in strains that overexpress heterologous thioesterases to divert flux towards FFAs. In *Y. lipolytica*, deleting *DGA1*, *DGA2*, *LRO1*, and *ARE1* combined with the overexpression of the thioesterase *TEII* from *Rattus norvegicus* facilitated the production of 3 g/L of exogenous FFAs (Ledesma-Amaro et al. 2016). We implemented a similar strategy in *P. pastoris*; however, strains with deletions in neutral lipid synthesis displayed reduced FA titers, productivity, and growth rates (Kobalter et al. 2023). A possible explanation for this finding could be that the deletions of acyltransferases led to a reduction in total FA flux through lipid bodies, and subsequently, fewer FFAs were released by TGLs, while the decreased acyl-CoA consumption led to feedback inhibition of preceding reactions. Additionally, neutral lipid storage might be required to balance FA metabolism in this species.

Aside from neutral lipids, polysaccharides and trehalose serve as energy reservoirs in yeast. It was demonstrated that the deletion of glycogen synthases improved TAG accumulation in a *Y. lipolytica* wild-type strain by over 60% (Bhutada et al. 2017). The same strategy was implemented in an extensively engineered *S. cerevisiae* strain, which elevated lipid contents by 5–10% (Arhar et al. 2021). Interestingly, the impairment of trehalose synthesis resulted in reduced TAG accumulation in *S. cerevisiae*.

At high-glucose concentrations, yeasts excrete significant amounts of fermentation products or other metabolites, which can reduce the availability of precursors for fatty acid synthesis. For example, *S. cerevisiae* produces ethanol, while oleaginous yeasts excrete citrate (Ledesma-Amaro et al. 2016; Yu et al. 2018). Therefore, interfering with fermentation processes presents an attractive target for increasing fatty acid yields. However, interventions in fermentative pathways can lead to growth arrests or slowed growth due to disturbances in redox cofactor balance, as seen with the deletion of *ADH1* in *S. cerevisiae*. Prolonged propagation of an *S. cerevisiae adh1∆* strain has been shown to partially recover from poor growth phenotypes (Li et al. 2014). Yu et al. effectively redirected carbon flux from ethanol fermentation to FFA production by using an adaptive laboratory evolution approach on a strain with deleted *PDC* (pyruvate decarboxylase) genes. This strategy successfully restored growth in the evolved strain (Yu et al. 2018). Critical mutations occurred in the *PYK1* gene, encoding the primary pyruvate kinase. These mutations reduced glycolytic flux, alleviating the need for NADH oxidation. Furthermore, enhanced NADPH synthesis through the pentose phosphate pathway was balanced by increased production of FAs.

In terms of TAG production, major competing processes include the synthesis of SEs and the breakdown of TAGs through lipases. To enhance TAG accumulation, reducing the rates of SE synthesis and TAG breakdown can be effective. For instance, deleting *TGL3* and *ARE2* in a TAG-accumulating *S. cerevisiae* strain increased TAG content by 34% and 25%, respectively. However, double deletions of both *TGL3* and *TGL4* or *ARE1* and *ARE2* genes resulted in reduced TAG accumulation and/or decreased strain fitness (Arhar et al. 2021). Lastly, the production of biomass competes with fatty acid synthesis. Excessive biomass accumulation is usually circumvented through nutrient limitations, such as controlling nitrogen or phosphate concentrations in the growth medium. However, concomitant downregulation or deletion of amino acid biosynthesis genes has been shown to further improve fatty acid yields (d’Espaux et al. 2017; Yu et al. 2018).

### Enhancing precursor availability—push engineering

Increasing the availability of acetyl-CoA, malonyl-CoA, and NADPH is a promising strategy to boost FA production. In nature, there are various pathways for acetyl-CoA synthesis, such as the yeast native PDH bypass and the ATP-citrate lyase shunt (ACL) found in oleaginous yeasts. Additionally, bacterial pathways like phosphoketolase/phosphotransacetylase (PK/PTA), pyruvate formate lyase (PFL), acetylating aldehyde dehydrogenase (AAD), and cytosolic pyruvate dehydrogenase (PDH) have been harnessed to boost the synthesis of fatty acids and other acetyl-CoA-derived products in yeast as reviewed by Arhar and Natter (2019) and Fernandez-Moya and Da Silva (2017). Furthermore, it was shown that mitochondrial acetyl-CoA can be shuttled to the cytosol using a carnitine acetyltransferase (Xu et al. 2016). These pathways differ in their acetyl-CoA, ATP, and NAD(P)H outputs and maximum theoretical yields (reviewed by van Rossum et al. 2016). The ACL shunt, which is regularly paired with a reduction in TCA flux through the downregulation of isocitrate dehydrogenases, and PK/PTA pathway are often preferred for their higher efficiency, whereas other pathways show certain limitations. For example, AAD and PDH produce cytosolic NADH, which cannot be used for fatty acid synthesis, contributing to an unfavorable redox balance. The PDH pathway also necessitates cytosolic lipoic acid production or lipoic acid supplementation. PFL is inhibited by molecular oxygen, which is suboptimal, as most bioprocesses for FA production are conducted under aerobic conditions and the PDH bypass is less energy efficient due to ATP conversion to AMP during acetyl activation. Nevertheless, all these strategies have been implemented in *S. cerevisiae* or in other yeasts to boost acetyl-CoA availability. In many cases, this has resulted in modest improvements in fatty acid titers, typically ranging from 5–30% (Gao et al. 2022; Tang et al. 2013; Zhou et al. 2016). However, certain studies have reported considerable improvements in product titers (1.8–2.3-fold) by implementing these strategies in a strain that had been optimized in downstream pathways (Feng et al. 2015; Xu et al. 2016). Consequently, acetyl-CoA engineering should be considered in later stages of strain modification, and its effectiveness will depend on the genetic background. Additionally, acetyl-CoA, ATP, and NADPH outputs can be balanced by combining different acetyl-CoA synthesizing pathways (van Rossum et al. 2016).

### Malonyl-CoA production

Malonyl-CoA synthesis is a critical rate-limiting step in fatty acid synthesis. As discussed previously, the activity of Acc1p is regulated through transcriptional and post-translational control. *ACC1* overexpression and deregulation through mutagenesis of phosphorylation sites (S659 A-S1157 A) have been shown to improve Acc1p activity by up to threefold (Shi et al. 2014). Implementing this strategy in a *S. cerevisiae* strain engineered for fatty acid ethyl ester (FAEE) synthesis considerably improved product titers. In another study, this approach led to a 2.6-fold improvement in fatty alcohol production in *S. cerevisiae* (d’Espaux et al. 2017). Additional putative phosphorylation sites on Acc1p, such as S1257, have been identified and could be mutated to further deregulate Acc1p activity (Arhar et al. 2021).

Since Snf1p responds to energy availability, it can be anticipated that the mutagenesis of Snf1p phosphorylation sites in Acc1p would not lead to considerable activity improvements in high-glucose cultivations, which are common for oleaginous yeasts (Qiao et al. 2015). Nevertheless, some level of regulation may still be maintained even at elevated glucose concentrations. This is supported by a study in *Lipomyces starkeyi*, where the deletion of *SNF1* enhanced lipid accumulation (Sato et al. 2024).

Overexpression and deregulation of Acc1p have also been successfully implemented in *Y. lipolytica* and *P. pastoris* (Cai et al. 2022; Kobalter et al. 2023; Tai and Stephanopoulos 2013). However, it is important to note that strains with elevated Acc1p activity may exhibit reduced growth and viability (Shi et al. 2014). This is likely due to the rapid consumption of acetyl-CoA or elevated malonyl-CoA levels. Additionally, enhanced Acc1p activity has been reported to inhibit inositol synthesis, which could explain reduced growth if inositol is not supplied in the growth medium (Shirra et al. 2001).

An alternative route for malonyl-CoA synthesis was recently introduced in *E. coli* by expressing a methyl-malonyl-CoA carboxyltransferase (MMC) from *Propionibacterium freudenreichii*. This enzyme naturally converts methyl-malonyl-CoA and pyruvate to propionyl-CoA and oxaloacetate, respectively, but it can also convert oxaloacetate and acetyl-CoA to pyruvate and malonyl-CoA (Park et al. 2022). MMC was co-expressed with a phosphoenolpyruvate carboxylase to ensure a sufficient supply of oxaloacetate. Implementing MMC in yeast could be valuable, as it is not subjected to the same regulatory constraints and could be efficiently coupled with the ATP-citrate lyase shunt, which provides acetyl-CoA and oxaloacetate while consuming pyruvate.

### Engineering the redox balance

Engineering the redox balance to enhance NADPH supply and optimize NADH consumption is a common strategy to increase fatty acid production in yeast. Many studies have focused on boosting NADPH supply by overexpressing endogenous pathways, such as the pentose phosphate pathway (PPP) or NADPH-dependent aldehyde dehydrogenase. Additionally, heterologous pathways have been utilized, including the overexpression of a NADPH-dependent glyceraldehyde 3-phosphate dehydrogenase, a NADH/NADPH transhydrogenase, or malic enzyme (native in many oleaginous yeasts) (reviewed in Fernandez-Moya and Da Silva 2017).

Specific examples of successful redox engineering include the expression of an NADP^+^ -dependent glyceraldehyde 3-phosphate dehydrogenase from *Clostridium acetobutylicum* and an NADP^+^ -dependent malic enzyme from *Mucor circinelloides*, respectively, which increased lipid yields by 20% and lipid titers by 28% in a *Y. lipolytica* strain engineered for TAG accumulation (Qiao et al. 2017). Additionally, overexpressing multiple PPP genes and downregulating glycolytic glucose- 6-phosphate isomerase through promoter exchange led to a 28% improvement in FFA production in *S. cerevisiae.* Concomitant downregulation or disruption of NADPH-consuming reactions, such as the one catalyzed by Gdh1p (NADP(+)-dependent glutamate dehydrogenase), can further support FA production (Yu et al. 2018). However, not all redox engineering approaches have been universally successful. The efficiency of these strategies can vary depending on the background strain and the existing NADPH availability (Arhar et al. 2021; Cai et al. 2022; d’Espaux et al. 2017).

### Engineering of fatty acid synthesis and desaturation—push–pull engineering

The production of FAs and FA-derived compounds can be further enhanced by overexpressing fatty acid synthase and ∆9-desaturase. Runguphan and Keasling overexpressed the endogenous *FAS1* and *FAS2* genes using the strong *TEF1* promoter in *S. cerevisiae*, which increased lipid content by 30% (Runguphan and Keasling 2014). Moreover, in *Y. lipolytica*, the overexpression of the endogenous ∆9-desaturase was shown to increase lipid titer, growth rate, and unsaturated FA content in a strain with enhanced Acc1p and Dga1p activity. This combination also provided resistance to high-glucose concentrations (150–300 g/L) (Qiao et al. 2015). The authors noted that the increased desaturase activity reduced levels of saturated acyl-CoAs, which specifically inactivate Acc1p, Fas1p, and Fas2p through allosteric control. Moreover, the shift towards unsaturated FAs improved acyl-CoA consumption, as acyltransferases preferentially incorporate unsaturated fatty acids, further alleviating regulatory constraints in preceding reactions.

Additionally, the regulation of fatty acid synthesis may be bypassed by expressing heterologous FAS systems. For instance, Eriksen et al. overexpressed a bacterial type I FAS (yeast-like) from *Brevibacterium ammoniagenes*, along with its phosphopantetheine transferase, to increase fatty acid ethyl ester (FAEE) production in an *S. cerevisiae* strain harboring a wax ester synthase (Eriksen et al. 2015). The resulting strain produced 6.3-fold more FAEEs than the basic strain.

*BaFAS* expression could complement FA production in a *S. cerevisiae fas1*Δ strain, restoring growth to near wild-type levels and considerably changing the intrinsic FA profile. Interestingly, the changes in FA composition were less pronounced when the endogenous FAS remained intact. Fernandez-Moya et al. replaced the endogenous FAS with a type II system from *E. coli* and co-expressed various thioesterases (Fernandez-Moya et al. 2015). This led to a 1.7-fold increase in FA titer, but reduced growth and specific productivity. Additionally, the modified strain displayed an altered FA composition with increased C14 and decreased C18 levels. This study highlighted the high innate activity of yeast FAS, but also pointed to potential opportunities for FA profile engineering by cytosolic expression of a type II system. Recently, reverse β-oxidation was implemented in yeast as a novel strategy for FA synthesis (Lian and Zhao 2015). This approach utilized acetyl-CoA and NADH directly for FA synthesis, avoiding the ATP cost associated with malonyl-CoA synthesis and potentially improving redox balance through NADH consumption. To enable reverse β-oxidation, the authors constitutively expressed a combination of endogenous and heterologous β-oxidation enzymes and targeted those to the cytosol by deleting signal peptides. Additionally, they co-expressed length-specific terminating enzymes, such as thioesterases and acyltransferases, to produce n-butanol, medium-chain fatty acids, and medium-chain ethyl esters. However, product titers remained relatively low at 20 mg/L n-butanol and 4–10 mg/L MCFAs, indicating the need for further optimization to achieve industrial relevance.

### Deregulation of fatty acid metabolism

Effective deregulation of fatty acid metabolism can be achieved by reducing acyl-CoA pools through overexpression of acyltransferases or deletion of acyl-CoA synthetases. Other strategies focus on manipulating transcription factors that regulate fatty acid synthesis. In *S. cerevisiae*, the transcription factors Ino2p and Ino4p form a complex that positively regulates the expression of *INO1*, which encodes inositol- 1-phosphate synthase, the key enzyme in inositol biosynthesis (Ambroziak and Henry 1994). Additionally, this complex activates genes involved in PL biosynthesis (Nikoloff et al. 1992), as well as *ACC1*, *FAS1*, and *FAS2* (Chirala 1992; Chirala et al. 1994). At low levels of PA in the endoplasmic reticulum, the negative regulator Opi1p translocates from the ER to the nucleus and antagonizes the activating function of the Ino2p-Ino4p complex, resulting in transcriptional repression of *INO1* and genes involved in PL and FA biosynthesis (Loewen et al. 2004).

D’Espaux et al. replaced the gene of the negative regulator *OPI1* with an overexpression cassette for the *INO2* gene and deleted the histone deacetylase gene *RPD3*, but neither modification yielded higher product titers (d’Espaux et al. 2017). Another study targeted different transcription repressors of *INO1* and the protein kinase gene *SNF1* for deletion in a strain overexpressing *ACC1* and a fatty acid reductase with the aim of achieving an increase in the expression of FA synthesis pathway genes. Only deleting *RPD3* and *MOT1* increased fatty alcohol production in this background (Feng et al. 2015). It could be anticipated that deregulation of the *INO1* network may be more effective in strains when *ACC1*, *FAS*, and *SCD*s are not overexpressed. The deletion of *SNF2*, encoding a component of the SWI/SNF chromatin remodeling complex, combined with overexpression of *DGA1* and expression of the selection marker *LEU2* (in an otherwise auxotrophic strain) facilitated the generation of an *S. cerevisiae* strain that accumulated 30% lipids of DCW (Kamisaka et al. 2007).

Strikingly, overexpression of an N-terminally truncated *DGA1* variant and downregulation of the histone acetyltransferase *ESA1* elevated TAG contents to 45% (Kamisaka et al. 2013). The impact of the *snf2* disruption was identified by screening a transposon knockout library using density gradient centrifugation to isolate strains with increased lipid content (lower specific density) (Kamisaka et al. 2006). In a different screening approach, Fei et al. evaluated *S. cerevisiae* mutant libraries for strains with supersized lipid droplets using microscopy (Fei et al. 2011). The deletion of one of the identified targets, *CKB1*, which encodes for a subunit of the casein kinase 2, in a TAG-accumulating *S. cerevisiae* strain, increased TAG content from 22% to almost 40%, while the deletion of another target, *SEI1* (Seipin), reduced TAG accumulation in this background (Arhar et al. 2021).

Strategies modifying regulatory networks have also been investigated in oleaginous yeast. The deletion of the AMP-dependent protein kinase Snf1p significantly improved lipid content in both *Y. lipolytica* and *L. starkeyi* (Sato et al. 2024; Seip et al. 2013). Co-overexpression of the transcription factor Spt23, which regulates desaturase expression in yeast, further increased lipid content in the *snf1*∆ *L. starkeyi* strain. Furthermore, expression of an Mga2p variant G643R in *Y. lipolytica* conferred increased lipid accumulation and higher unsaturated FA content, particularly C18:1 and C16:1. This highlights the importance of desaturase overexpression for lipid accumulation. Transcriptional analysis revealed an upregulation of genes involved in glycolysis, acetyl-CoA synthesis, fatty acid production, and desaturation in the strain expressing the Mga2p variant (Liu et al. 2015).

Given that various engineering strategies like increasing precursor supply, overexpressing fatty acid synthase, desaturase or terminal enzymes, and deregulating FA metabolism have been shown to increase FA titers and/or specific productivity, it can be concluded that there is not a single rate-limiting step in fatty acid synthesis; rather, the overall synthesis rate is governed by the interplay of multiple reactions. Furthermore, since the turnover rates of individual reactions vary among hosts and strains, the effectiveness of a given engineering strategy is often dependent on the genetic background.

## Fatty acid, fatty acid derivative, and triglyceride products

**Fig. 2:**
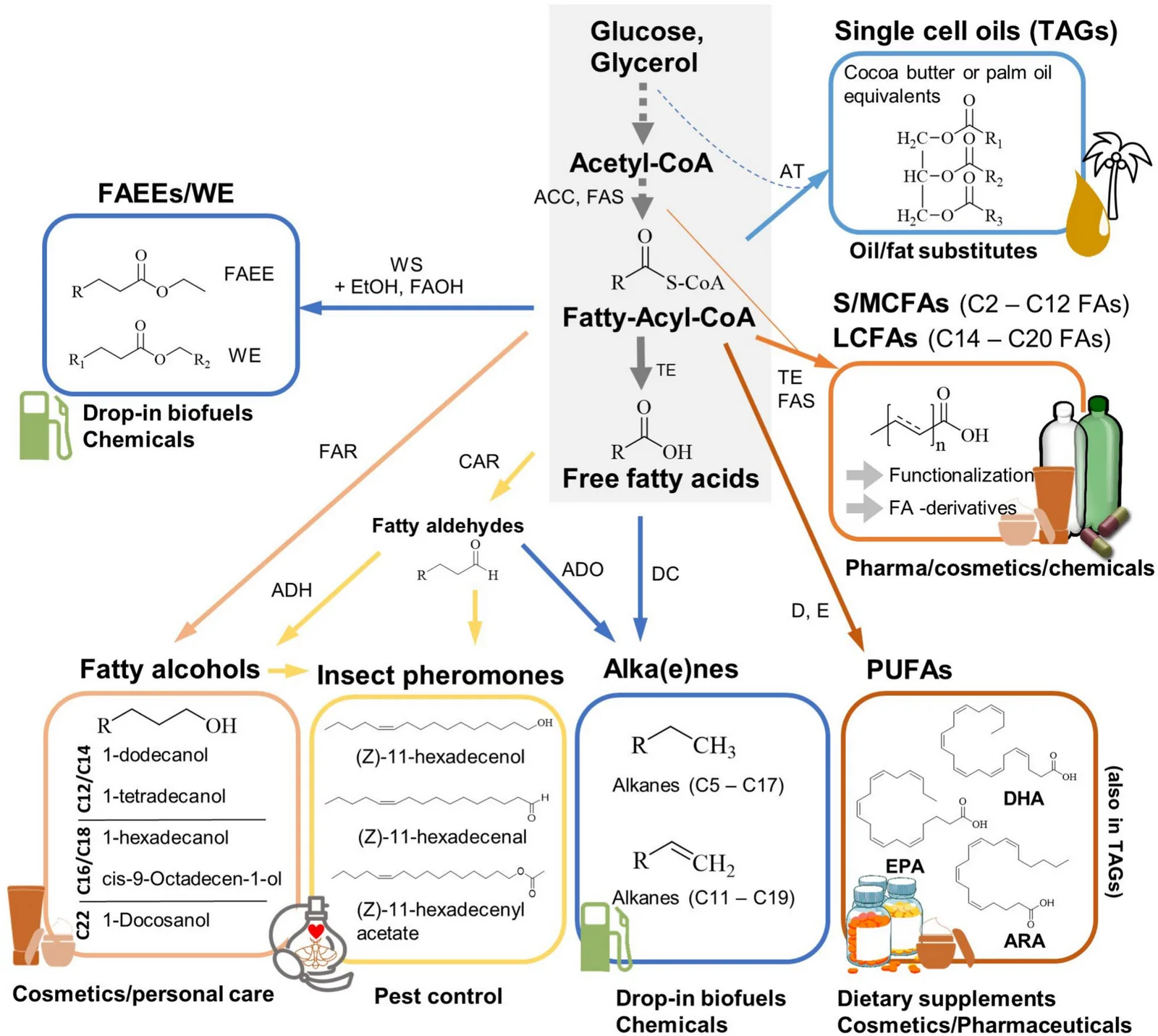
Value-added products derived from fatty acids and derivatives, which can be synthesized by yeast cell factories with tuned fatty acid profiles. The simplified metabolic pathway for fatty acid synthesis and major terminal enzymes are depicted. *ACC*, Acetyl-CoA carboxylase; *FAS*, fatty acid synthase; *TE*, thioesterase; *WS*, wax ester synthase; *FAR*, fatty acid reductase; *CAR*, carboxylic acid reductase; *ADH*, alcohol dehydrogenase; *ADO*, aldehyde deformylating oxidase; *DC*, decarboxylase; *D*, desaturase; *E*, elongase; *AT* acyltransferase; FAOH, fatty alcohols

There are various value-added FAs, FA derivatives, and TAG products that can be produced by engineering FA compositions in yeast. These include SFAs and MCFAs, long mono- and polyunsaturated FAs, VPUFAs, fatty alcohols, FA ethyl esters, wax esters, and alka(e)nes as well as cocoa butter and palm oil substitutes. The following section will discuss engineering strategies to synthesize these products in modified yeasts and provide suggestions for host selection to streamline profile modifications. Figure 2 depicts relevant products and their simplified synthesis routes. Table 1 presents FA profiles of popular oleaginous and non-oleaginous yeasts, and Table 2 lists examples for the production of FAs, FA derivatives, and TAGs with altered FA composition using engineered yeast strains.

**Table 1:** Fatty acid compositions of industrially relevant oleaginous and non-oleaginous yeasts; lipid content given as % of CDW

Species and products	Fatty acid distribution %							Lipid content %	Ref
	C16:0	C16:1	C18:0	C18:1	C18:2	C18:3	other		
Oleaginous yeasts									
*Yarrowia lipolytica*	14	14	1	51	19	0	1	18	Kolouchová et al. (2015)
*Rhodosporidium toruloides*	20	1	15	47	13	4	1	68	Li et al. (2007)
*Cutaneotrichosporon oleaginosus*	30	1	6	48	10	< 1	5	43	Wei et al. (2017)
*Lipomyces starkeyi*	32	2	10	48	4	< 1	4	18	Wei et al. (2017)
*Rhodotorula glutinis*	15	7	9	64	2	0	3	65	Taskin et al. (2016)
Non-oleaginous yeasts									
*Saccharomyces cerevisiae*	12	39	4	40	-	-	4	4	Kolouchová et al. (2015)
*Pichia pastoris*	5	6	1	46	30	11	3	n/d	Kobalter et al. (2023)
*Schizosaccharomyces pombe*	11	3	5	78	0	n/d	3	5	Yazawa et al. (2012)
*Kluyveromyces lactis*	15	24	3	20	30	7	1	n/d	Micolonghi et al. (2012)
Products									
Cocoa butter	26	0	35	35	3	0	1		Abeln and Chuck (2021)
Palm oil	44	0	4	40	10	0	2		Abeln and Chuck (2021)

**Table 2:** Examples for the synthesis of FAs, FA derivatives, or TAG products with modified chain length, degree of saturation, and FA profiles in yeast

Products	Chain length	Sp	Modifications, cultivation conditions ^a^	Titer, FA content	Cultivation	Ref
MCFA	C6–C12	*Sc*	↑AcCoA ScFAS*-AcTesA MvFAS*-AcTesA TPO1-F322L* faa1*∆ *faa4*∆ ACC1** AcCoA dodecane overlay	2.87 g/L	FBB	Zhu et al. (2020)
MCFA in TAGs	C14	*Yl*	***↑EgDGAT FAS-I1220 W**** dgat1*∆ *dgat2*∆ *pox1*∆	45% C14 in TAG	SF	Rigouin et al. (2018)
POA in TAG	C16:1	*Ss*	***↑9D**** ↑DGAT AcCoA ↑JEN12*	7.4 g/L, 24.9% of TFA	FBB	Qian et al. (2024)
POA in TAG	C16:1	*Sc*	***↑’MGA2 elo2***∆ *KCS ***improved medium composition, fermentation temperature**	6.5 g/L, 50% of TFA	FBB	Zhang et al. (2022a, b)
EPA	C20:5	*Yl*	***↑6D ↑18/20E ↑5D ↑17D**** ↑12D ↑16/18E*	40% TFA	n/d	Zhu et al. (2010)
DHA	C22:6	*Yl*	***↑6D ↑18/20E ↑5D ↑17D ↑20/22E ↑4D**** ↑12D ↑16/18E*	6% of TFA	n/d	Zhu et al. (2010)
EPA	C20:5	*Yl*	***↑8D ↑5D ↑17D ↑18/20E**** ↑12D ↑16/18E ↑CPT1 pex10*∆	56.5% of TFA	SF	Xue et al. (2013)
DHA	C22:6	*Ls*	***↑15D,*** (rest n/d)	17.4% of TFA	FBB	Salunke et al. (2015)
DHA	C22:6, C20:4	*Yl*	***↑PKS***	350 mg/L, 16.8% of TFA	FBB	Gemperlein et al. (2019)
Fatty alcohols	C16–C18	*Sc*	***↑FAR**** gdh1*∆ *dga1*∆ ↑ACC1** ↑9D hfd1∆ *adh6*∆	6 g/L	FBB	d’Espaux et al. (2017)
Fatty alcohols	C16–C18	*Sc*	***↑CAR ↑ADH5**** ↑RtFAS ↑ACC1 pox1*∆ *↑EcTesA ↑AcCoA faa1*∆ *faa4*∆ *hfd1*∆	1.5 g/L	FBB	Zhou et al. (2016)
Alkanes/alkenes	C16–C18	*Sc*	***↑CAR ↑ADO**** adh5*∆ *↑RtFAS ↑ACC1 pox1*∆ *↑EcTesA ↑AcCoA faa1*∆ *faa4*∆	0.82 mg/L	FBB	Zhou et al. (2016)
FAEE	C16–C18	*Rt*	***↑AbWS-G355I****, ethanol addition*	10 g/L	FBB	Zhang et al. (2021a, b)
Insect pheromone	Z11-C16:1-OH	*Yl*	***↑11D ↑FAR FAS-I1220 F**** pex10*∆ *hfd1*∆ *hfd4*∆ *fao1*∆* ↓GPAT*	2.57 g/L	FBB	Holkenbrink et al. (2020)
Insect pheromones	Z7-C12:1-OH Z9-C12:1-OH	*Yl*	***↑*partial β-oxidation*, ↑FAR,* C14 feed**	0.4–0.5 mg/L	24 WP	Petkevicius et al. (2022)
CBE	C16–C18	*Ac*	***9D***, n/d (random mutagenesis)	45.7% L DCW, CBL n/d	BB	Ykema et al. (1990)
CBE	C16–C18	*To*	**Nitrogen limitation**, wild-type	42.8% L DCW, 27.8% pCBL	SF	Wei et al. (2017)
CBE	C16–C18	*Rt*	**Nitrogen limitation**, wild-type	16.6% L DCW, 31.9% pCBL	SF	Wei et al. (2017)
CBE	C16–C18	*Sc*	***↑Ch9D 9 d***∆ ***(or ↑D9)*** ↑16/18E ACC1**	3–8% L DCW, 10–22% pCBL	SF	Bergenholm et al. (2018)

^a^Major profile defining modifications and terminal enzymes in bold; Z11-C16-OH, (Z)-hexadec- 11-en- 1-ol, Z7-C12-OH, (Z)-dodec- 7-en- 1-ol; Z9-C12-OH, (Z)-dodec- 7-en- 1-ol; ↑, gene overexpressed; #*D*, desaturase—# distance of introduced double bond from carboxy terminus; #/#*E*, elongase—extends from #1 to #2; *DGA* or *DGAT*, diacylglycerol acyltransferase; *FAR*, fatty acid reductase (acyl-CoA dependent); *GDH1*, glutamate dehydrogenase 1; *ACC1*, acetyl-CoA carboxylase; ACC1**, *ACC1*^S659A S1157A^; *CAR*, carboxylic acid reductase; *ADH*, alcohol dehydrogenase; ‘*TesA*, thioesterase, *FAS*, fatty acid synthase; ScFAS*, *ScFAS*^G1250S M1251W^; MvFAS*, *MvFAS*^G2684S M2685W^; *POX1*, acyl-CoA oxidase; *FAA*, acyl-CoA synthetase; AcCoA, increased acetyl-CoA supply, *JEN12*, mitochondrial carboxylate transporter; *HFD1 - 4*, fatty aldehyde dehydrogenase; *FAO1*, fatty alcohol oxidase; GPAT, glycerol- 3-phosphate acyltransferase, *PEX10*, peroxisomal protein importer; *CPT1*, choline phosphotransferase; ‘*MGA2*, truncated transcription factor; *Sc, Saccharomyces cerevisiae; Yl, Yarrowia lipolytica; Rt, Rhodosporidium toruloides; Ls, Lipomyces starkeyi; Ac, Apiotrichum curvatum; To, (Neo)Trichosporon oleaginosus, Ss, Scheffersomyces segobiensis*; TFA, total fatty acids; pCBL, potential cocoa butter lipids; FBB, fed-batch bioreactor; BB, batch bioreactor; SF shake flasks; 24 WP, 24-well plate

### Short- and medium-chain fatty acids

SFAs and MCFAs are highly valued feedstocks in the chemical industry, utilized for producing transportation fuels, surfactants, cosmetics, and plastics (Sarria et al. 2017; Zhu et al. 2020). However, natural pathways for S/MCFAs are scarce because biological membranes predominantly use fatty acids with chain lengths of 16 to 18 carbon atoms. To address this limitation, most production approaches focus on terminating native FA synthesis at specific chain lengths by introducing heterologous thioesterases (or other terminal enzymes) with high selectivity for S/MCFAs.

Heterologous thioesterases can interact efficiently with the dissociated bacterial type II FAS and to a lesser extent with animal type I FAS (Fernandez-Moya et al. 2015; Leber and Da Silva 2014). However, they cannot directly interact with the ACP domain of fungal type I FAS due to its tightly packed and rigid structure (Jenni et al. 2007; Zhu et al. 2017). Consequently, specific thioesterases are incorporated into the fungal FAS complex to facilitate the release of FFAs during de novo fatty acid synthesis (Zhu et al. 2017). Alternative strategies include the mutagenesis of FAS structures to prematurely release FAs or the replacement or co-expression of the endogenous FAS with heterologous FAS systems from animal or bacterial origins (type II) to enhance interactions with thioesterases (Fernandez-Moya et al. 2015; Gajewski et al. 2017; Leber and Da Silva 2014). Thioesterase incorporation was demonstrated for the thioesterase *‘Ac*TesA from *Actinobacter baylyi*, with specificity for S/MCFAs, which was integrated into the *S. cerevisiae* and *R. toruloides* type I FASs adjacent to the ACP domains. These FAS variants were expressed in a FAS null *S. cerevisiae* background, resulting in strains that produced approximately twice the amount of S/MCFAs compared to strains co-expressing the wild-type FAS and an independent ‘*Ac*TesA. This demonstrates that incorporating the thioesterase into the reaction chamber is crucial for enhanced S/MCFA production (Zhu et al. 2017). Moreover, structure-guided engineering of the *S. cerevisiae* FAS was conducted to identify mutations that promote SCFA release, unraveling hotspots in the KS, MPT, and AT domains. The amino acid exchanges G1250S and M1251 W in the KS domain create steric hindrance for SCFA extension, promoting S/MCFA release, while the R1834 K exchange in the MPT domain reduces affinity for malonyl, promoting early acyl termination. Furthermore, the I306 A exchange in the AT domain increases acetyl loading efficiency, leading to elevated SCFA synthesis (Gajewski et al. 2017). Combining the KS domain mutations with the incorporation of a thioesterase further improved S/MCFA synthesis (Zhu et al. 2017).

Since S/MCFAs are toxic to cells, leading to detrimental effects on cellular membranes and intrinsic pH levels, strain engineering has also focused on increasing resistance to these compounds. Zhu et al. conducted directed evolution on the MCFA exporter Tpo1p and used adaptive laboratory evolution by cultivating cells in a C8-containing medium to enhance MCFA resistance. Overexpressing the engineered FAS in the evolved strains, combined with increased precursor supply, deletions in β-oxidation and fatty acid activation pathways, and the addition of a dodecane overlay for in situ product removal, facilitated the production of 1.4 g/L MCFAs in shake flasks and 2.87 g/L in fed-batch cultivations, which are the highest titers reported in literature (Table 2) (Zhu et al. 2020).

In another study, the human fatty acid synthase was co-expressed with heterologous phosphopantetheine transferases (*AcpS* from *E. coli* or *Sfp* from *B. subtilis*) and short-chain-specific thioesterases (*CpFATB1* from *C. palustris* or *TEII* from rat mammary gland) in *S. cerevisiae*. Furthermore, the thioesterase domain of the human FAS was replaced with the short-chain-specific thioesterases, resulting in the secretion of 80–110 mg/L C8, representing a four- to ninefold improvement compared to strains separately expressing these enzymes (Leber and Da Silva 2014).

FAS engineering for MCFA production has also been performed in *Y. lipolytica* (Xu et al. 2016). Here, the MPT domain of the endogenous FAS was replaced with different thioesterases that exerted distinct acyl-length specificities, leading to a sixfold increase in C12 fatty acids with the thioesterase domain from *Umbellularia californica*. Furthermore, by expressing a medium-chain-specific diacylglycerol acyltransferase from *Elaeis guineensis* in a background with deleted diacylglycerol acyltransferases and a *Yl*FAS2^I1220 W^ mutant, C14 FA species (C14:0 and C14:1) accumulated up to 45% of total lipids in TAGs (Rigouin et al. 2018).

### Long-chain fatty acids

Mono- and polyunsaturated LCFAs (C16–C18) are essential for human health, exhibiting protective properties against cardiovascular diseases, metabolic disorders, and cancer (López-Miranda et al. 2006). The most investigated members include oleic acid, linoleic acid (particularly conjugated linoleic acid), and γ-linolenic acid (GLA). Their production in yeast has been reviewed by Cao et al. (2022) and Szczepańska et al. (2022).

A long-chain fatty acid that has only recently gained attention due to its low abundance in most nutritional fats is palmitoleic acid (POA). POA offers several health benefits, including protective effects against metabolic disorders and anti-inflammatory properties (Bermúdez et al. 2022). Furthermore, POA is also used in cosmetics and as an antibacterial agent (Watanabe et al. 2019). Currently, POA is primarily sourced from macadamia nuts (Hu et al. 2019) and sea buckthorn seeds (Solà Marsiñach and Cuenca 2019), respectively, containing roughly 20% and 30% POA of total fatty acids. Biotechnological production using yeasts offers an eco-friendly and more effective alternative to these plant-based sources. POA typically constitutes a small fraction of the total fatty acids in most oleaginous and non-oleaginous yeasts, ranging from 5 to 10%. However, certain exceptions exist, such as *Kluyveromyces lactis* (25% POA) (Micolonghi et al. 2012), *S. cerevisiae* (30–40% POA), and *Vanderwaltozyma polyspora* (former: *Kluyveromyces polysporus*) (35–60% POA) (Kolouchová et al. 2015), presenting optimal hosts for the production of this FA. Additionally, the FA profiles of other yeasts can be engineered to increase POA titers. For example, Qian et al. engineered the recently discovered oleaginous yeast *Scheffersomyces segobiensis* by introducing the *S. cerevisiae* ∆9-desaturase (Qian et al. 2024). Additional overexpression of diacylglycerol acyltransferase and PK/PTA pathway allowed the production of 29.6 g/L lipids (24.9% POA) in bioreactors. POA content increased from approximately 10% in the wild-type strain to 25% in the engineered strain, when cultivated with elevated dissolved oxygen concentrations.

Furthermore, *S. cerevisiae* was engineered for elevated POA production by constitutively expressing the desaturase regulator Mga2 and deleting fatty acid elongases responsible for extending POA. The authors deleted the C-terminal ER anchor domain of Mga2p, generating a free activator that continuously induced the expression of *OLE1* and other fatty acid pathway genes. Additional deletion of *ELO2* and heterologous expression of a plant elongase (keto acyl-CoA synthase from *Cardamine graeca*) to maintain VLCFA synthesis increased POA content from ~ 32% in the wild-type to 58.5% (Zhang et al. 2022a, b). In a follow-up study, the same strain was used for POA production from corn stover hydrolysate. Optimization of process parameters, including changes in amino acid composition, carbon-to-nitrogen ratio, and reduced temperature during the production phase, resulted in the production of 6.5 g/L POA and a POA content > 50% of all FAs (Li et al. 2023). In contrast, unmodified *K. polysporus* produced 1.06 g/L microbial lipids with a POA content of 61.3% of total FAs in complex medium with 1% glucose (patent: JP3090810B2). Notably, there are no reports on genetic engineering of* K. polysporus,* which could hinder strain development for commercial production. We recently engineered the methylotrophic yeast *P. pastoris* for the secretion of free POA, by overexpressing heterologous thioesterases, ∆9-desaturases, and fatty acid synthases. The highest improvements in POA content were obtained by expressing a C16-specific ∆9-desaturase from *Mus musculus* and replacing the endogenous FAS with the FAS from *S. cerevisiae*. POA content increased from 6% in the wild-type to 22% in the final strain (Kobalter et al. 2023).

### Very long-chain polyunsaturated fatty acids (VPUFAs)

VPUFAs such as eicosapentaenoic acid (EPA, C20:5 ω3), docosahexaenoic acid (DHA, C22:6 ω3), and arachidonic acid (ARA, C20:4 ω6) are highly valued for their nutritional benefits. These fatty acids have demonstrated protective properties against cancer, cardiovascular diseases, and diabetes (Shahidi and Ambigaipalan 2018). Additionally, VPUFAs are integral structural components of cell membranes and precursors to important signaling compounds like prostacyclins, leukotrienes, and prostaglandins (Funk 2001; Ma et al. 2004). VPUFAs are naturally found in the fat of deepwater fish and commercial production primarily relies on fish oil extraction, though certain products based on fats from microalgae and fungi are already available (Szczepańska et al. 2022). Marine microorganisms naturally produce VPUFAs via either an aerobic desaturase-elongase-dependent pathway or an anaerobic polyketide synthase (PKS) pathway (Gemperlein et al. 2014, 2019). The desaturase-elongase pathway can be further categorized into a ∆− 6 pathway and a ∆− 9 pathway, with the key difference being the C18 elongases that convert either ∆6- or ∆9-FAs, yet both pathways utilize oleic acid or linoleic acid as initial substrates (Xue et al. 2013; Zhu et al. 2010). Consequently, optimal host strains for VPUFA production should possess high C18 content, naturally produce linoleic acid (LA), and accumulate large amounts of lipids, traits common in many oleaginous yeasts such as *Y. lipolytica* and *R. toruloides*. High innate PUFA content, as often found in *Rhodotorula* species, could also be beneficial to prevent membrane distortion caused by elevated unsaturated FA levels.

Efficient EPA production has been achieved by introducing the ∆6 and ∆9 pathways in *Y. lipolytica*. The ∆6 pathway was introduced by integrating multiple copies of Δ12-desaturase, C16/18 elongase (to enhance LA production), Δ6-desaturase, C18/20 elongase (Δ6 specific type), Δ5-desaturase, and Δ17-desaturase. The resulting strain produced 40% EPA and 21% γ-linolenic acid in total FAs (Zhu et al. 2010). However, the authors found high GLA contents, particularly in the TAG fraction, which can be unfavorable for certain food and supplement formulations. It should be noted that the heterologous desaturases convert acyl chains bound to PLs, whereas the elongases extend fatty acids in the acyl-CoA form. Consequently, efficient VPUFA synthesis requires the exchange of fatty acid intermediates contained in PL/TAG and acyl-CoA pools. Such an exchange would include the release of FAs from PLs or TAGs through lipases, followed by reactivation with CoA, elongation, incorporation into PLs, and desaturation. The authors noted that GLA accumulation was due to insufficient mobilization of FAs from stored TAGs rather than the low activity of the 18/20 elongase, as externally provided FFAs were efficiently extended by the heterologous elongases. Additional integration of a C20/22-elongase and a Δ4-desaturase in the EPA-producing strain enabled the synthesis of 6% DHA.

In a follow-up study, the ∆9 pathway was introduced in *Y. lipolytica* through random genomic integration of multiple expression cassettes, including various desaturases, C16/18-elongases, a ∆9 elongase, and a choline phosphotransferase. The localization of randomly integrated expression cassettes revealed the disruption of the *PEX10* gene, encoding a peroxisomal protein importer, which significantly contributed to the VPUFA accumulation. The best strain produced 56.6% EPA of total FAs and the lipid content reached 30% of the dry cell mass (Xue et al. 2013). This strain was further developed into a commercial EPA production system by DuPont (Xie et al. 2015). *Y. lipolytica* was also employed for the synthesis of ARA via the desaturase-elongase pathway, achieving 0.76% of total FAs (Liu et al. 2019). Aside from *Y. lipolytica*, other oleaginous yeast species have been explored for VPUFA production. For example, *Lipomyces starkeyi* was used to produce DHA using the elongase-desaturase approach, resulting in DHA comprising 17.4% of total FAs (Salunke et al. 2015).

Lastly, the PKS pathway has been harnessed to produce PUFAs in yeast. PKS form multidomain complexes analogous to FAS, with mostly the same catalytic domains (KS, KR, DH and ER) and an independent PPT domain. Previously, gene clusters of dedicated VPUFA-producing PKSs had been identified in marine and soil microorganisms (Gemperlein et al. 2014). A major advantage of the PKS-based VPUFA synthesis over the conventional route is the reduced NADPH demand (14 NADPH for DHA via PKS compared to 26 in the desaturase-elongase pathway). PKSs incorporate double bonds by omitting the enoyl-reduction step at certain chain positions followed by an isomerization reaction (trans to cis), thereby saving NADPH for saturated bond synthesis and the subsequent desaturation. The expression of DHA- or ARA-producing myxobacterial PKS in a mostly unmodified *Y. lipolytica* strain enabled the production of 350 mg/L DHA (16.8% of total FAs) and 12 mg/L ARA (Gemperlein et al. 2019). It remains to be seen whether these enzymes will perform accordingly in heavily engineered strains that accumulate high lipid contents.

### Fatty alcohols, fatty acid ethyl/methyl esters, alkanes, and alkenes

Fatty alcohols, alkanes, alkenes, and fatty acid ethyl/methyl esters (FAMEs/FAEEs) are highly desired compounds due to their applications as drop-in fuels, lubricants, surfactants, cosmetics, and flavor and fragrance compounds (Sarria et al. 2017; Zhang et al. 2022a, b). Directly synthesizing these FA derivatives eliminates the need for chemical transesterification or derivatization of fatty acids or TAGs. FA derivatives can be produced in vivo from either free fatty acids or fatty acyl-CoAs (Fig. 2). Fatty alcohol synthesis from FFAs involves two reduction steps. First, FFAs are converted to fatty aldehydes by carboxylic acid reductases, followed by another reduction of fatty aldehydes to fatty alcohols catalyzed by alcohol dehydrogenases. An alternative, possibly more efficient route is the direct conversion of acyl-CoAs to fatty alcohols using acyl-CoA reductases (FARs). Overexpression of a *M. musculus FAR* in an extensively engineered *S. cerevisiae* strain led to the production of 6 g/L fatty alcohols in fed-batch bioreactor cultivations (d’Espaux et al. 2017). In comparison, the FFA-based two-step reaction achieved a maximum titer of 1.5 g/L in a similar cultivation setup (Zhou et al. 2016). It should be noted that strain backgrounds used in these studies differed, making a direct comparison difficult.

Efficient fatty alcohol production has also been achieved in oleaginous yeasts like *R. toruloides*. The expression of the acyl-CoA reductases from *Marinobacter aquaeolei* coupled with optimization of fermentation conditions allowed the production of 8 g/L of C16–C18 fatty alcohols in fed-batch mode (Fillet et al. 2015). It should be noted that the highest fatty alcohol titer in literature was obtained in *E. coli* through extensive metabolic remodeling, achieving 12.5 g/L (Fatma et al. 2018). Among their various applications, fatty alcohols also serve as insect pheromones that can be applied for species-specific pest control for the protection of plant crops (see below).

Alkane production from FFAs requires the activities of a carboxylic acid reductase and an aldehyde deformylating oxidase to reduce the carboxy moiety to an aldehyde followed by the release of a 1-carbon unit (Zhou et al. 2016). Alternatively, FFAs are directly converted to alkanes/alkenes by light- or hydrogen peroxide-dependent decarboxylases (Rude et al. 2011; Sorigué et al. 2017). Most biotechnological approaches for alkane production are limited by low activities of the terminal enzymes, as often indicated by the accumulation of FA intermediates (Zhou et al. 2016). Consequently, the highest titers for alkane production in yeast are in the lower mg/L range (Zhou et al. 2016), while the highest titer obtained in literature resides at 5.2 g/L achieved with the bacterium *R. opacus* (Kim et al. 2019). Due to the low in vivo activity of decarboxylases in yeast, it could be valuable to first accumulate TAG followed by cell lysis and enzyme-based conversion of TAGs to FFAs and alkanes. Jiang and coworkers have established an efficient enzymatic cascade for the conversion of TAGs to alpha olefins (Jiang et al. 2020).

FAEEs/FAMEs are synthesized through wax ester synthases that catalyze the esterification of acyl-CoAs with alcohols of different lengths, also enabling the production of wax esters (Kalscheuer et al. 2006; Shi et al. 2012). Ethanol is highly abundant in *S. cerevisiae*, and heterologous overexpression of different wax ester synthases allowed the production of up to 0.5 g/L FAEEs with extensive engineering efforts (Yu et al. 2012a, b), yet most studies only reported titers in the low mg/L range (Zhang et al. 2022a, b). Astonishingly, through heterologous wax ester synthase expression coupled with supplementation of ethanol and tuned process engineering, Zhang and coworkers could demonstrate the production of 10 g/L FAEEs in *R. toruloides* with a yield of 0.086 g/g (Zhang et al. 2021a, b). Even though most of the carbon was channeled towards biomass and neutral lipids, this study nevertheless demonstrated the high potential of *R. toruloides* for FAEE production. The highest FAEE titer among microbes was achieved with the bacterium *R. opacus* (21.3 g/L FAEEs; fed-batch) (Kim et al. 2019).

Current efforts in metabolic engineering are also aiming to produce FA derivatives from S/MCFAs, which are often preferred over the long-chain counterparts for various applications (Zhu et al. 2020). This requires fatty acid profile remodeling coupled with the engineering of derivatizing enzymes to tailor their substrate specificity towards shorter acyl chains. Recently, the carboxylic acid reductase from *Mycobacterium marinum* was engineered for improved activity on MCFA substrates through structure-guided design and directed evolution. A *S. cerevisiae* strain expressing the evolved variant produced 2.8-fold more medium-chain fatty alcohols than a strain harboring the wild-type variant (Hu et al. 2020).

### Insect pheromones

Synthetic insect pheromones provide an opportunity for eco-friendly and highly specific pest control in crops. The release of species-specific pheromones into fields leads to mating disruption or mating delay of certain pests, while other potentially beneficial insects remain unharmed (Reddy and Guerrero 2010). Additionally, insect pheromones are non-toxic and do not pollute the environment. So far, only insect pheromones from moths and some other lepidopterans have been extensively investigated, as these present the major crop pests. Most insect pheromones are fatty acid derivatives that can be categorized into type I, type II, and miscellaneous type pheromones, respectively, representing 75%, 15%, and 10% of currently known compounds (Ando et al. 2004). Type I pheromones are comprised of C10 to C18 fatty aldehydes, fatty alcohols, or fatty alcohol acetate esters with one or two double bonds (Fig. 2). Type II pheromones are C17 to C25 hydrocarbons with two to three double bonds, which are often epoxy-functionalized. Lastly, the miscellaneous group contains short-chain secondary alcohols, long-chain ketones, epoxides, and methyl-branched compounds (Ando et al. 2004; Ando and Yamamoto 2020). Since type I pheromones comprise the largest class among insect pheromones, this group has attracted the most attention in the research community. Carbon backbones for type I pheromones are produced through fatty acid de novo synthesis, followed by desaturation of acyl-CoAs and conversion to fatty alcohols by acyl-CoA-dependent fatty acid reductases. Fatty alcohols can then be further converted to fatty aldehydes or acetate esters by alcohol dehydrogenases and alcohol/acetate transferases or through chemical synthesis. Positions of double bonds, length specificities of FARs, and the types of terminal enzymes are species-specific. Thus far, all bioprocesses have focused on the fermentative production of fatty alcohols and employed chemical synthesis methods to generate the final derivatives. However, in situ conversion could also be envisioned, which could involve the deletion of alcohol dehydrogenases or the expression of acetate transferases. Insect type I pheromone production in yeast has recently been reviewed by Zhang et al. (2021a, b), particularly highlighting the potential of *S. cerevisiae* and *Y. lipolytica* for this purpose. Holkenbrink and colleagues produced (Z)-hexadec- 11-en- 1-ol (Z11-C16:OH) in *Y. lipolytica*—a precursor for *H. armigera* (cotton bollworm) and other pest pheromones, at 2.57 g/L in bioreactor cultivations (Holkenbrink et al. 2020) (Table 2). Strain engineering involved blocking acyl-CoA and fatty alcohol degradation, as well as overexpression of an insect ∆11-desaturase and FAR. In a recent study, desaturase and FAR expression were combined with partial β-oxidation, to generate medium-chain pheromones (Z)− 7-dodecenol (Z7 - 12:OH), (Z)− 9-dodecenol (Z9 - 12:OH), and other truncated pheromone precursors from supplemented methyl myristate in *Y. lipolytica* (Table 2). By examining different acyl-CoA oxidases and targeting the FAR to the peroxisome, the medium-chain pheromones were successfully produced, albeit at lower titers (Petkevicius et al. 2022). Elucidating pheromone production pathways in many more insects will foster enzyme discovery for the production of short-chain fatty acids and FA derivatives in other hosts and will likely expand current product spectra.

### Production of single-cell oils for food applications

Single-cell oils can potentially substitute for food oils or fats. Currently, the application of SCOs appears to be most plausible for high-value fats like cocoa butter or fats with improved nutritional value (i.e., high PUFA fats, see above). The production of cocoa butter equivalents (CBEs) using oleaginous yeasts has been extensively researched over the past decades (Papanikolaou and Aggelis 2011), almost leading to the commercialization of a CBE product. However, declining cocoa butter (CB) prices after 1990 eventually led to the cancelation of the project. Cocoa butter is primarily used in chocolate and cosmetics manufacturing and has a high saturated fatty acid content of 55–65% (Table 1) (Papanikolaou and Aggelis 2011). The relative position and type of fatty acid in the glycerol backbone of TAG also significantly impact cocoa butter properties (Lipp and Anklam 1998). The main TAGs in cocoa butter are 1,3-dipalmitoyl- 2-oleoyl-glycerol (POP), 1-palmitoyl- 2-oleoyl- 3-stearoyl-glycerol (POS), and 1,3-distearoyl- 2-oleoyl-glycerol (SOS), collectively referred to as cocoa butter-like lipids (CBLs). Thus, oleic acid is always found in the sn- 2 position, while palmitic or stearic acid occupies the other positions (Papanikolaou and Aggelis 2011). Current CBE production methods use lipase-based re-/inter-esterification of mixed cheap oil sources to generate the required FA and TAG profiles (Fig. 2). Oleaginous yeasts are appealing hosts for CBE production due to their similar FA profiles, predominantly consisting of saturated and monounsaturated fatty acids with C16 or C18 carbon atoms. However, these yeasts typically have higher oleic acid and lower stearic acid content compared to cocoa butter, with an unsaturated FA content of about 65%. Consequently, engineering efforts and process optimization have focused on reducing the activity of the endogenous ∆9-desaturase to modify the FA profile. Process optimizations, such as cultivating at lower dissolved oxygen levels, lower temperatures, and using desaturase inhibitors and nutrient limitations, have shown some improvements in the FA profile (Davies et al. 1990; Moreton and Clode 1988; Wu et al. 2011, 2010). Furthermore, strains of *Apiotrichum curvatum* (*Oleaginosum curvatum*) were subjected to ethyl methanesulfonate mutagenesis and selected for diminished ∆9-desaturase activity. Reverted mutants with reduced desaturase activity resembled the FA profile of cocoa butter (Ykema et al. 1990), laying the basis for an attempted commercialization of CBE. In a recent study, process conditions were optimized for CBL production in *R. toruloides*, leading to the accumulation of 55–60% lipids of DCW with 27% CBLs. Additionally, most other TAGs contained oleic acid in the sn- 2 position (Yang et al. 2024). In *Y. lipolytica*, the endogenous *OLE1* gene was replaced with the variants from *R. toruloides* or *Gloeophyllum trabeum* to shift the FA profile towards that of CB, resulting in reduced C16:1 content. Promoter exchange of the heterologous desaturases further increased the stearic acid content, though the strains exhibited reduced growth and lipid content, possibly due to altered FA compositions in phospholipids (Konzock et al. 2022).

CBE production has also been investigated in *S. cerevisiae*. Since this yeast has a high C16:1 content, of which cocoa butter is mostly devoid, the researchers aimed to reduce C16:1 content while increasing C16:0, C18:0, and C18:1. This was achieved by downregulating or exchanging the endogenous *OLE1* gene with a desaturase from *Calanus hyperboreus* showing low activity on C16:0 (Bergenholm et al. 2018). Additionally, the *ACC1*^S659A−S1157A^ variant and the endogenous *ELO1* gene were overexpressed to push FA production and aid in the extension of palmitic acid to stearic acid. These modifications nearly eliminated C16:1 content and elevated other fatty acids, yet the strain showed reduced growth. Downregulation of the endogenous *OLE1* gene also reduced TAG content, which could be compensated for by expressing the Acc1 variant. The best engineered strains displayed increased CBL amounts of up to 20%. Furthermore, CBL contents were increased in *S. cerevisiae* by expressing cocoa tree acyltransferase genes (glycerol- 3-phosphate acyltransferase (GPAT), lysophospholipid acyltransferase (LPAT), and diacylglycerol acyltransferase (DGAT)) by replacing one of the endogenous counterparts, which raised CBLs from below 1% in the wild-type to 6% in the engineered strains and slightly increased lipid accumulation (Wei et al. 2018). In a comparative study, six yeast species were evaluated for growth rate and lipid accumulation, particularly CBE, in nitrogen-limited medium. While *S. cerevisiae*, *Rhodosporidium graminis*, and *Y. lipolytica* accumulated CBLs in the range of 4–10%, *Trichosporon oleaginosus* (*Cutaneotrichosporon oleaginosum*), *L. starkeyi*, and *R. toruloides* contained 28–32% CBLs (Wei et al. 2017). Among these, *T. oleaginosus* and *R. toruloides* exhibited the highest growth rates and *T. oleaginosus* exerted the highest TAG content, making these two yeasts optimal hosts for CBE production. It should be noted that SOS contents are comparably low (below 2.5%) in most yeast species, suggesting that engineering efforts should focus on enhancing SOS content (Wang et al. 2020).

Palm oil is the cheapest and most produced plant fat globally at over 70 million tons per year and finds application in food, cosmetics, and biofuel sectors (Szczepańska et al. 2022). The increasing demand has led to extensive deforestation, raising sustainability concerns. Palm oil’s FA composition (Table 1) partially matches the lipids produced by oleaginous yeasts like *L. starkeyi* or *R. glutinis*. However, microbial production of palm oil substitutes is currently elusive due to the low selling price of palm oil. A recent techno-economic analysis indicated that microbial palm oil substitute production could only be feasible if valuable byproducts are synthesized and the generated yeast biomass is sold (Karamerou et al. 2021). Thus, oil production could be coupled for example with recombinant protein secretion, while the residual biomass is to be used for animal feed.

Yeast biotechnology holds potential for producing high-value fats and fatty acids that closely resemble those found in dairy, eggs, meat, and human milk. A promising application of microbial fats is in infant nutrition, where researchers seek to replicate the complex lipid profile of human breast milk. Human milk fat features a unique TAG structure, with palmitic acid predominantly occupying the sn- 2 position, while unsaturated FAs are positioned at sn- 1 and sn- 3. This specific configuration improves nutrient absorption in infants. In contrast, conventional infant formula fats, typically derived from plant oils, lack this arrangement, resulting in lower absorption efficiency (Jiang et al. 2022). Oleaginous yeasts provide a sustainable alternative for lipid production, yet their native TAG composition also excludes palmitic acid from the sn- 2 position. To address this limitation, Bhutada et al. (2022) expressed lysophosphatidic acid acyltransferases with high specificity for palmitoyl-CoA in *Y. lipolytica*, achieving over 60% incorporation of palmitic acid at the sn- 2 position. This advancement enhances the potential of yeast-derived fats as functional substitutes for human milk fat in infant nutrition.

To the best of our knowledge, no studies have yet explored the production of dairy, egg, or meat fat substitutes using yeasts. However, the growing demand for vegan alternatives, coupled with advancements in cost-effective biotechnological production, could enable future developments in this field.

## Conclusion and future perspectives

Conventional and oleaginous yeasts are excellent producers of microbial oils and FA-derived products due to their high lipid accumulation and innate high activity of FA synthesis (Arhar et al. 2021; Babau et al. 2013; Qiao et al. 2017; Yu et al. 2018). However, only a few yeast oil and FA derivative products have been commercialized, which are mostly restricted to high-value products. This limitation is due to the low costs of fossil oil-derived chemicals and high-yield plant oils like palm fat, alongside the relatively high requirements for biotechnological production. Nevertheless, there are ongoing efforts to make biotechnological production of low-cost lipids economically feasible. Promising strategies to reduce costs involve substituting sugar-based feedstock for lignocellulose hydrolysates or waste streams (Angerbauer et al. 2008; Slininger et al. 2016) and conducting unsterile fermentations under extreme conditions (Moustogianni et al. 2015). Furthermore, efficient downstream processing can be facilitated by secreting lipid products directly into the fermentation medium (Cajka et al. 2016) or by implementing cell wall-degrading enzymes for in situ cell lysis.

Aside from economic considerations, engineering microbes for optimized fatty acid profiles present additional challenges. Extensive modifications of FA compositions have been reported to impair growth and reduce lipid content (Kobalter et al. 2023; Konzock et al. 2022). Altered FA compositions in PLs disturb membrane functions, especially when high saturated FA contents are observed. Potential solutions include constantly remodeling FAs in phospholipids by exchanging saturated for unsaturated FAs, implementing adapted laboratory evolution to evolve strains for growth with modified profiles, or decoupling growth from production. The synthesis of MCFAs leads to cytotoxic effects, as MCFAs cause membrane leakage and alter cytosolic pH levels. Thus, there is the need to engineer yeast strains for increased resistance to these compounds. The toxicity of MCFAs could be partially mitigated by sequestrating MCFAs with FFA-binding proteins. Another possible solution could involve the efficient export of MCFAs. Zhu and coworkers engineered the MCFA exporter Tpo1 through directed evolution, which increased growth in medium containing C10 FAs and improved extracellular MCFA titers of a MCFA-secreting *S. cerevisiae* strain (Zhu et al. 2020). Aside from this example, however, the knowledge on FA exporters is currently limited (Salvador López and Van Bogaert 2021). Furthermore, little is known about how released FFAs migrate to the plasma membrane for subsequent export.

In regards to producing FA derivatives, the transport of FFAs and acyl-CoAs to derivatizing enzymes could also be limiting, as FFAs and long acyl-CoAs are poorly soluble in water and were shown to adhere to proteins and intrinsic membranes due to their hydrophobic nature (Sumper and Träuble 1973). In general, activities for FA derivatizing enzymes appear to be lower in yeast than in bacteria (Zhou et al. 2018). Therefore, identifying new FA-modifying enzymes that are highly active in yeast or engineering known ones for enhanced activity will be pivotal to establish efficient synthesis of FA derivatives in yeast.

Moreover, specific fat substitutes, like CBE, require particular FA distributions on the glycerol backbone of TAGs, mostly of the saturated–unsaturated-saturated (SUS) type (Lipp and Anklam 1998; Papanikolaou and Aggelis 2011). Since yeasts possess low quantities of these fats (Wei et al. 2017), it will be essential to discover novel acyltransferases capable of producing the required TAG species.

Despite ongoing challenges, various studies have demonstrated the outstanding potential of yeasts for the synthesis of lipid-derived products, including the production of TAGs above 100 g/L (Babau et al. 2013), FFAs at 33.4 g/L (Yu et al. 2018), fatty alcohols at 6.0 g/L (d’Espaux et al. 2017), and S/MCFAs at 2.87 g/L (Zhu et al. 2020), among others. Moreover, the literature has presented various successful examples of engineering FA compositions in yeasts (Holkenbrink et al. 2020; Xue et al. 2013; Zhang et al. 2022a, b; Zhu et al. 2020). A fine-tuned interplay between FA synthesizing, modifying, and transferring enzymes is necessary to achieve custom FA compositions. FAs of shorter chain length can be produced by incorporating length-specific thioesterases into type I FAS or exploiting β-oxidation to truncate acyl chains. Lastly, the length specificities of terminating enzymes like thioesterases, acyltransferase, or fatty acid reductases are crucial for altering the compositions of stored or secreted FAs. New insights into advanced bioprocess development, FA metabolism, and its regulation will pave the way towards the commercialization of additional high-value FA products and low-cost fat substitutes.

## Abbreviations

FA: Fatty acid
FFA: Free fatty acid
TAG: Triacylglycerol
MCFA: Medium-chain fatty acid
LCFA: Long-chain fatty acid
VLCFA: Very long-chain fatty acid
VPUFA: Very long-chain polyunsaturated fatty acid
DHA: Docosahexaenoic acid
EPA: Eicosapentaenoic acid
ARA: Arachidonic acid
PA: Phosphatidic acid
PL: Phospholipid
SE: Sterol ester
DAG: Diacylglycerol
FAEE: Fatty acid ethyl ester
FAME: Fatty acid methyl ester
